# Mutations in the voltage-gated sodium channel associated with permethrin resistance in *Rhipicephalus linnaei* populations in Thailand

**DOI:** 10.1038/s41598-025-91600-0

**Published:** 2025-03-02

**Authors:** Ratree Takhampunya, Wasinee Ngonsawan, Asma Longkunan, Siriporn Phasomkusolsil, Sommai Promsathaporn, Bousaraporn Tippayachai, Jira Sakolvaree, Alyssa N. Mann, Erica J. Lindroth

**Affiliations:** Department of Entomology, Walter Reed Army Institute of Research-Armed Forces Research Institute of Medical Sciences, Bangkok, Thailand

**Keywords:** Permethrin resistance, *Rhipicephalus linnaei*, Voltage-gated sodium channel, Larval packet test, Haplotype analysis, Genetic association study, Genetic markers

## Abstract

Insecticide resistance is a serious threat to vector control programs worldwide. Therefore, it is crucial to monitor the development of resistance in vector populations. *Rhipicephalus linnaei* (Audouin, 1826) is a cosmopolitan tick and a vector of medically important pathogens. We conducted a comprehensive investigation of permethrin resistance in larvae of *Rh. linnaei* populations across Thailand by comparing phenotypic resistance with tick genotypes, focusing on mutations in Domain II and III of the voltage-gated sodium channel gene. Results showed that larvae obtained from engorged female tick populations in Thailand have developed resistance to permethrin, with levels varying by location. Resistance ratios ranged from 1 to 56 when compared to the least susceptible local population. Genotyping identified mutations at positions 190 (c.190C > A and c.190C > G) and 2134 (c.2134T > C) in Domain II and Domain III, respectively, which are correlated with phenotypic resistance. We identified new alleles c.190CG and c.190AG in highly resistant populations from Phasichareon, Bangkok, and Chonburi provinces. This study provides the first evidence, to our knowledge, of permethrin resistance in *Rh. linnaei* ticks in Thailand. Elevated levels of permethrin resistance in *Rh. linnaei* populations across Thailand indicate that veterinarians and farmers should consider tick control products with alternative modes of action.

## Introduction

The prevalence of tick-borne diseases in Thailand has been investigated^1–4^, but only a handful of studies have been done on acaricide susceptibility to the major classes of chemicals currently in the market. A wide range of products, including synthetic pyrethroids, such as cypermethrin and chlorpyrifos cypermethrin, have been imported into Thailand for the control of insect pests in agriculture, horticulture, and forestry (https://data.go.th/dataset/importinsecvol, data collected from 2005 to 2021). Fipronil has been imported since 2020 due to the high demand for residential termite control and in agricultural use^5^. In Thailand, commonly used pyrethroid-based products include Bull’s Eye (Cypermethrin), Molical 25 (Cypermethrin), Cyper 10% Line (Cypermethrin), and Saima 35 (Cypermethrin) for agricultural use; DELGUARD (Deltamethrin) 50, 100, 150 EC, CYPERGUARD (Cypermethrin) 10, 25 EC for household use; and Frontline Tri-ACT (Fipronil + Permethrin) and Effitix (Fipronil + Permethrin) for companion animals. Additionally, permethrin is the most common active ingredient (AI) used as a textile treatment and has been used to treat United States and partner nation military uniforms to prevent insect bites, including mosquitoes and ticks, for nearly 30 years^6^. The extensive use of permethrin in agricultural and urban pest control and the cross-resistance between pyrethroids and other organochlorines^7 ^ has resulted in widespread resistance to permethrin in mosquitoes and ticks^7–10^. Pesticide resistance may occur through several modes of actions or a combination of actions. Examples of metabolic detoxification include elevated expression of esterase activity in *Rhipicephalus sanguineus*^11^, P450 cytochrome oxidase enzymes in *Rhipicephalus microplus*^12^, and glutathione-*S*-transferase in *Rh. sanguineus* and *Rh. microplus*^13,14.^ Additionally, point mutations resulting in insensitivity of the target protein such as the voltage-gated sodium channel gene in *Rh. sanguineus* to permethrin^11,15,16^, can also lead to resistance.

While there are numerous reports on permethrin resistance in mosquitoes, the study of permethrin resistance in ticks, particularly *Rh. sanguineus* and *Rh. linnaei*, still requires extensive research^11,15,17–23^. The majority of available data come from *Rhipicephalus* (*Boophilus*) *microplus*, an important pest of livestock, affecting production and causing economic losses worldwide^24–29^. Recently, *Rh. sanguineus* resistance to permethrin has also been reported globally^11,15,18,19^. In Thailand, Siriporn et al.^30^ recently reported the status of *Rh. sanguineus* s.l. resistance to commonly used acaricides (ivermectin and fipronil) in companion pets in Maha Sarakham province, Thailand. In this study, a low level of resistance to ivermectin and moderate-to-severe resistance to fipronil were observed^31^. Still, data on acaricide resistance in *Rh. linnaei* ticks in Thailand is limited and requires more attention due to its impact on humans and companion pets.

Most pathogens carried by *Rh. sanguineus* and *Rh. linnaei* impact the health of companion animals, such as *Ehrlichia canis*, *Babesia* spp., *Hepatozoon canis*, and *Anaplasma platys*^32–34^. However, these ticks also transmit life-threatening pathogens tohumans,  such as *Rickettsia rickettsii*, the aetiological agent of Rocky Mountain spotted fever (RMSF), *Rickettsia conorii*, and *Rickettsia massiliae*^35–39^. In addition, climate change may affect the host preference of the brown dog tick, leading to a higher risk of infection^39,40^. The *Rh. sanguienus* s. l. complex is distributed globally^41^ and generally separated into two distinct clades, tropical and temperate, according to their morphological, biological, and genetic difference^42,43^. The tropical lineage has been reclassified as *Rhipicephalus linnaei* (Audouin, 1826)^44,45^. The difference in *Rh. sanguineus* clades could influence their vector competency for some pathogens, especially *R. rickettsii* and *R. conorii*^19^. *Rhipicephalus sanguineus* is a three-host tick but preferentially infests domestic animals, especially domestic canines. This behavior, in turn, makes *Rh. sanguineus *and *Rh. linnaei * highly adaptable to human households and communities, thus increasing their exposure to acaricides used both in the environment and on domestic animals themselves.

This study evaluated the status of acaricide resistance in larvae obtained from engorged female *Rh. linnaei* collected throughout Thailand. The Food and Agricultural Organization Larval Packet Test (LPT)^46^, was conducted to assess phenotypic resistance in tick populations. Genotyping of populations phenotypically resistant and susceptible to permethrin was conducted by investigating mutations occurring in the voltage-gated sodium channel (*vgsc*) gene, particularly in domain II and III, which contain knockdown resistance (kdr) mutations common to several vectors, including mosquitoes. A polymerase chain reaction (PCR)-restriction fragment length polymorphism (RFLP) assay was developed and validated using previously identified genotypes by DNA sequencing. The newly developed assay accurately matched the genotypes identified through DNA sequencing results. The correlation between resistant phenotypes and genotypes was determined, and resistance markers were identified for *Rh. linnaei* populations in Thailand. The resistance markers revealed in this study may be useful for evaluating the status of permethrin resistance in *Rh. linnaei* ticks across Southeast Asia.

## Results

### Phenotypic assessment of tick populations in Thailand for permethrin resistance

#### Lethal concentration values and resistance ratio for all field collected ticks

Local *Rh. linnaei* larval ticks obtained from engorged females were analyzed for their susceptibility or resistance to permethrin using the LPT. Engorged female ticks were collected from one site in each of 14 provinces, and from two sites each in Bangkok and Chonburi provinces, and assessed for permethrin resistance. Phenotyping results indicated that larval ticks have established varying levels of resistance compared with those from females collected in Lamphun province (Table 1 and Supplementary Table S1 online). Larval ticks from Lamphun province displayed the most susceptibility to permethrin and thus were used as a baseline for comparison. Resistance ratio levels of *Rh. linnaei* populations in Thailand ranged from 1.00 to 56.00, with ticks from Phasichareon, Bangkok (RR = 56.00, 95%CI 36.00, 87.00) presenting the highest, and those from Lamphun, the lowest value (RR = 1.00, 95%CI 0.62, 1.60). The lethal concentration 50 (LC-50) of the *Rh. sanguienus* colony population (OSU-USA) was 0.02% (w/v) (95%CI 0.00, 0.09), while the LC-50 for Thai populations ranged from 0.21% to 12.00% w/v (95%CI 0.10, 47.00), as detailed in Table 1.

**Table 1:** Toxicity of permethrin to *Rhipicephalus linnaei* larvae obtained from engorged female tick populations collected from 16 provinces in Thailand as determined using the FAO larval packet test and their resistance allele frequencies (RAF) of three SNPs occurred at Domain II and Domain III of voltage-gated sodium channel gene.

Locations	GPS coordinates	Number of larvae (No. of replicates)	LC_50_ (min, max)	Resistance Ratio (95% CI)		Resistance allele frequency (RAF)					
				*Rh. sanguineus* as baseline	*Rh. linnaei* (Lamphun) as baseline	Nonsynnonymous mutation				Synonymous mutation	
						N (473)	c.190C > A	c.215G > T	c.2134 T > C	N (192)	c.2166 T > C
*Rh. sanguineus* (temperate lineage)	NA	1825 (3)	0.02 (0, 0.09)	1 (0.87, 1.15)	–	10​	5.00​	5.00​	0	10	0
Ayuthaya	14.359522,100.468141	2219 (4)	0.36 (0.24, 0.49)	22 (14, 35)​	1.73 (0.97, 3.08)	16​	31.30​	34.40​	0	7	50.00
Bangkok (Talingchan)	13.774576,100.454502	2371 (4)	1.29 (1.03, 1.74)	80 (17, 362)​	6.16 (1.26, 30.00)	35​	38.57​	38.57​	4.29​	0	0
Bangkok-2 (Phasichareon)	13.70567,100.436632	1849 (3)	12.00 (5.12, 48.00)	701 (512, 961)​	56.00 (36.00, 87.00)	31​	100**​	71.00	0	29	68.97
Chachoengsao	13.676509,101.829725	1599 (3)	3.57 (2.48, 5.80)	237 (152, 304)​	17.00 (11.00, 27.00)	30​	41.70​	41.70​	21.70​	7	35.71
Chantaburi	12.798287,102.270591	1844 (2)	4.17 (3.15, 5.97)	251 (176, 360)​	20.00 (12.00, 32.00)	21​	19.05​	28.57​	7.14​	21	30.95
Chiangrai	20.095829,99.784805	1365 (3)	0.33 (0.26, 0.42)	20 (12, 35)​	1.60 (0.84, 3.05)	10​	0	20.00​	0	10	50.00
Chumphon	10.641169,99.238549	2129 (3)	5.68 (2.58, 25.00)	342 (249, 471)​	27.00 (17.00, 43.00)	36​	52.78​	25.00​	20.83​	12	37.50
Krabi	8.242495,98.907946	1724 (3)	3.33 (2.14, 6.25)	201 (155, 261)​	16.00 (11.00, 24.00)	10​	0	0	35.00​	10	0
Lamphun	18.182539,99.003476	2283 (4)	0.21 (0.10, 0.35)	13 (9, 18)​	1.00 (0.62, 1.60)	32​	0	6.25​	0	-	-
Lopburi	14.982613,100.923111	2230 (5)	0.60 (0.49, 0.71)	47 (14, 91)​	2.85 (1.07, 7.56)	30​	41.70​	51.70​	11.70​	3	33.33
Nakhonprathom	13.773549,100.313707	1614 (4)	1.92 (1.10, 3.93)	116 (62, 217)​	9.15 (4.52, 18.00)	16​	68.75​	68.75​	31.30​	6	41.67
Petchabun	16.572714,100.98949	1508 (4)	2.80 (1.96, 4.43)	169 (110, 260)​	13.00 (7.83, 23.00)	28​	42.86​	42.86​	35.71​	10	45.00
Phayao	19.201325,100.061173	2470 (4)	1.00 (0.89, 1.14)	61 (0.00, 2.75e + 32)​	4.79 (0, 4.96E + 31)	30​	23.33​	23.33​	16.67​	2	50.00
Prachinburi	14.159327,101.523804	1472 (3)	4.65 (3.26, 7.36)	298 (199, 395)​	22.00 (14.00, 35.00)	35​	42.86​	30.00​	27.14​	-	-
Ratchaburi	13.507891,99.865883	1642 (4)	0.29 (0.15, 0.45)	18 (11, 26)​	1.39 (0.83, 2.31)	30​	0	53.30​	0	2	50.00
Suratthani	9.059651,99.256014	2710 (5)	0.52 (0.36, 0.72)	32 (17, 60)​	2.50 (1.23, 5.08)	10​	40.00​	30.00​	25.00​	1	50.00
Chonburi-1 (Bang Lamung)	12.899615,100.865698	1019 (2)	NA	–	–	30​	51.70*​	48.30	48.30	29	50.00
Chonburi-2 (Ban Chang)	12.695174,100.985838	1047 (2)	NA	–	–	33​	90.90**​	80.30	3.00	33	80.30

LC = Lethal Concentration (% w/v). All mortality data was corrected using Abbott’s formula, (*), new alleles (c.190AA and c.190CG) were observed at position 190, (**), new alleles (c.190AA and c.190AG) were detected for position190 and both are homozygous resistant genotypes, (NA), larvae from this location were strongly resistant to permethrin, even at the highest concentration used (10% w/v) in the LPT assay: Chonburi-1 (Bang Lamung) = 0.9% mortality at 10% w/v permethrin, Chonburi-2 (Ban Chang) = 12% mortality at 10% w/v permethrin.

**Fig. 1:**
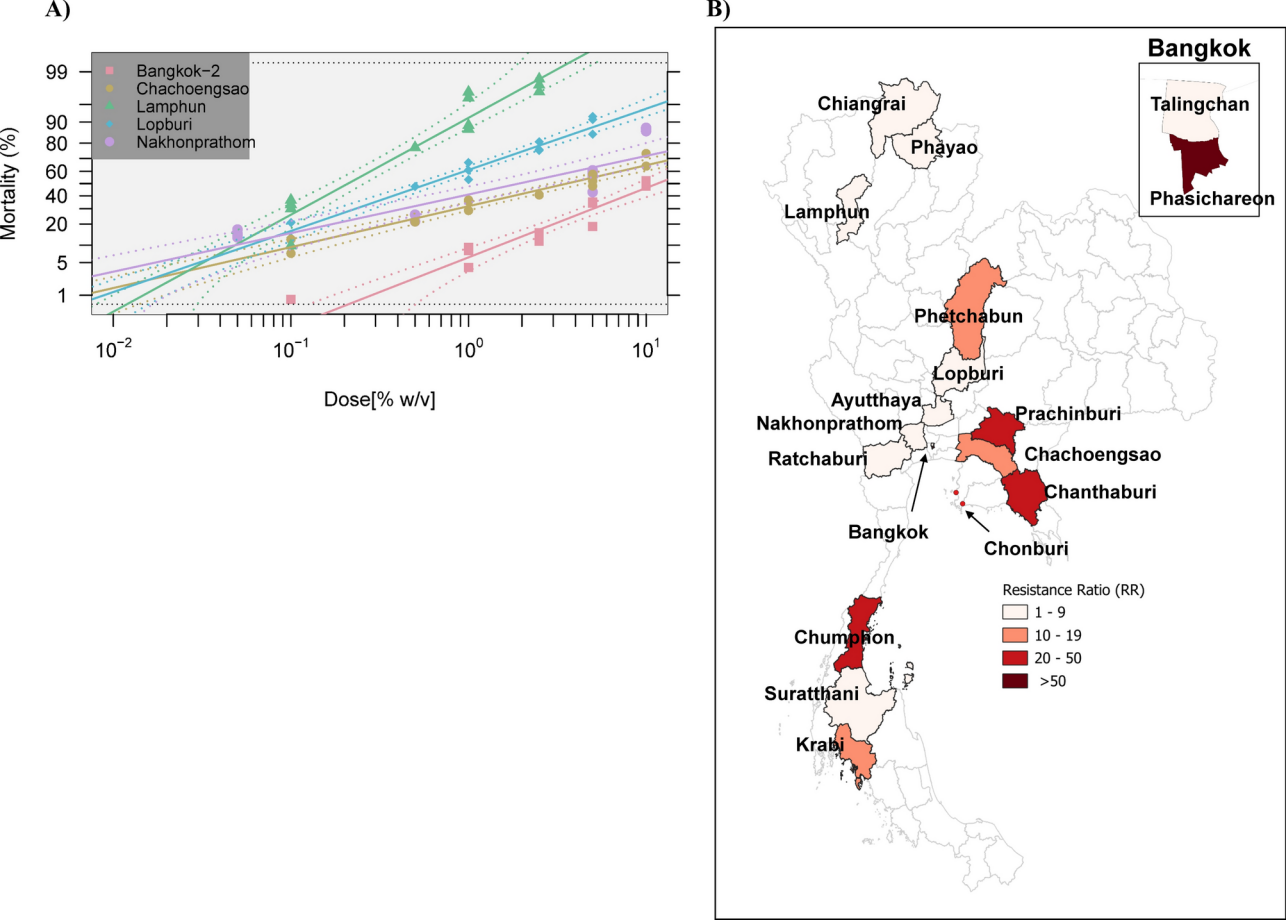
Probit graphs were generated using the BioRssays package in RStudio, version 2024.9.0.375 (**A**). This graph depicts linear relationships between probit-transformed mortality rates and the log-dose of permethrin (%, w/v) for different tick populations. Representative tick populations with different resistance levels are shown (RR = 1.00 Lamphun, RR = 2.85 Lopburi, RR = 9.15 Nakhonprathom, RR = 17.00 Chachoengsao, RR = 56.00 Bangkok-2). A heat map demonstrates the overall permethrin resistance status of *Rh. linnaei* ticks in Thailand (**B**). GPS coordinates for sampled locations are presented in Table 1.

Figure 1A presents probit analyses derived from LPT data, including five populations of *Rh. linnaei* in Thailand, representing the resistance ratio levels detected in this study. Probit analyses of all strains along with the comparison population from Oklahoma State University (OSU), USA can be found in Supplementary Fig. S2 online. Figure 1B is a heatmap depicting the permethrin resistance status of *Rh. linnaei* populations in Thailand, with strong resistance observed in the Bangkok metropolitan area as well as several eastern and southern provinces.

The Chi-square goodness of fit test for populations from Lamphun and Nakhon Prathom failed, therefore these populations were excluded from the Likelihood Ratio Test (LRT). The similarity of the mortality-dose regression among different populations was tested using the LRT as described in the Methods section. The model test revealed that the majority of populations showed significant differences in the magnitude of the response and/or slope (*p*-value < 2.2e-16) (see Supplementary Table S2 online). However, some populations exhibited significant similarities in the mortality-dose regression. These populations exhibited similar resistance levels, such as among tick populations collected from Ayutthaya, Chiangrai, and Ratchaburi, or populations from Bangkok and Phayao, Lopburi and Surathani (see Table 1 and Supplementary Table S2 online for a more detailed pairwise comparison).

### Genotypic assessment of tick populations in Thailand for permethrin resistance

#### Genotyping of ticks using DNA sequencing

**Fig. 2:**
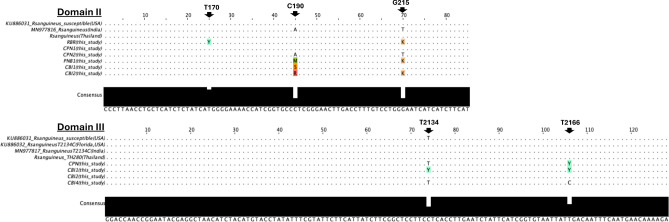
Alignment of *vgsc* gene Domain II and III shows consensus sequences and mutations occurring in Domain II (at positions 170, 190, 215) and in Domain III (at positions 2134 and 2166). Ambiguous sequences are indicated as follows: Y = T/C, K = G/T, M = A/C, R = A/G, S = C/G.

DNA sequence alignment of Domain II and III of the voltage-gated sodium channel (*vgsc*) gene is shown in Fig. 2. As indicated by arrows, the observed mutations were located at positions 170, 190, 215 in Domain II, and at positions 2134, and 2166 in Domain III, according to the nucleotide sequence position of *Rh. microplus* (Accession number AF134216). Any ambiguous sequences are highlighted in color. Three mutations in Domain II (c.170T > C, c.190C > A or c.190C > G, and c.215G > T) and two mutations in Domain III (c.2134 T > C and c.2166 T > C) were identified. Mutations (c.170T > A, c.170T > C, c.190C > A, and c.215G > T) in Domain II and one mutation (c.2134 T > C) in Domain III have been reported in *Rh. microplus* and *Rh. sanguineus* previously^15,21^. These mutations result in changes in amino acids, specifically Leucine (Leu) to Isoleucine (Ile) (c.190C > A mutation), Leucine (Leu) to Glycine (Gly) (c.190C > G mutation), Glycine (Gly) to Valine (Val) (c.215G > T mutation), and Phenylalanine (Phe) to Leucine (Leu) (c.2134T > C mutation). This study identified a new homozygous resistant genotype (RR), A and G nucleotides at position 190 (c.190AG, resulting in an amino acid change to Isoleucine and Glycine), and the mutation c.2166T > C. The mutation c.2166T > C is synonymous and does not result in an amino acid change.

The clarity of sequence chromatograms enabled us to identify multiple nucleotides at each single nucleotide polymorphism (SNP) position. Chromatograms detailing SNPs found in Domains II and III are presented in the Supplementary Fig. S4 online. A heterozygous genotype is characterized by dual peaks—indicative of two nucleotides at the same position—as opposed to a single peak characteristic of homozygous susceptible or resistant genotypes. The presence of a super-*kdr* mutation (c.170T > C) was observed in 7 out of 210 (3.3%) tested larvae from two locations (Chumphon and Ratchaburi provinces) (see Supplementary Table S4 online). This particular SNP was only found in 6 out of 17 larvae (Chumphon) and 1 out of 4 larvae (Ratchaburi) surveyed, therefore we did not include this mutation in the downstream analysis. Additionally, the c.2166T > C SNP occurred at a high rate (48.7%) and was found in nearly all locations, thus was not included in the downstream analysis. Moreover, detailed genotype comparisons between engorged females and their offspring are accessible in the supplementary material (Table S5 online).

#### Development of a PCR-RFLP assay for rapid SNP detection in domains II and III

Before the in-house PCR–RFLP assay was developed, DNA sequencing was employed to identify SNPs in domain II and III of the *vgsc* gene. Samples from five provinces (N = 80) were initially sequenced. These known sequences comprising all genotypes were used as references for PCR–RFLP assay development. The technique was used to specifically detect SNPs, namely c.190C and c.215G in Domain II, and c.2134 T in Domain III. Unique genotypes found in these ticks, confirmed by DNA sequencing, were used to evaluate whether selected restriction endonuclease (RE) enzymes produced distinct RFLP patterns (see Supplementary Fig. S6 online). The results revealed that the PCR product from Domain II (167 bp), when cut with RE BsaJI, was able to differentiate among nine genotypes resulting from one SNP or the combination of two SNPs (c.190C > A and c.215G > T). For Domain III, RE MboII was used to cut the PCR product (135 bp), which generated three distinct genotypes (SS:c.2134TT, RR:c.2134CC, RS:c.2134TC) within this domain (Supplementary Fig. S5 online).

The PCR–RFLP assay was validated with a selected set of previously identified samples, by DNA sequencing, comprising different genotypes (Table 2). For Domain II, out of 48 sequenced samples, the PCR–RFLP assay accurately detected the two mutations in 46 samples (95.83%). Only two samples (positions 190 SS and 215 SS) were misidentified as RS in the PCR–RFLP results. However, upon reviewing the raw sequencing chromatograms of these two samples, small peaks nearly at the level of background noise were observed (Supplementary Fig. S7 online). For Domain III, the PCR–RFLP assay correctly identified all 28 samples to match the sequencing results (28/28, 100%). For highly resistant populations found in two locations in Chonburi and Phasichareon, Bangkok, all samples underwent sequencing. The PCR–RFLP was employed to evaluate a larger sample size from each population and location (Supplementary Table S6 online).

**Table 2:** Comparison of DNA sequencing and PCR–RFLP assay for identifying SNPs and mutations in Domains II and III of the voltage-gated sodium channel (*vgsc)* gene in *Rhipicephalus linnaei* from Thailand.

Domain of *vgsc* gene	Genotypes	Genotyping methods performed on individual tick	
		DNA sequencing	PCR–RFLP
II (c.190C > A/c.215G > T) (N = 48)	SS*/SS	14*	13
	RS*/SS	6	7*
	SS/RS	5	5
	SS/RR	3	3
	RR/SS^#^	3^#^	2
	RR/RS^#^	3	4^#^
	RR/RR	2	2
	RS/RR	2	2
	RS/RS	10	10
III (c.2134 T > C) (N = 28)	SS	14	14
	RS	11	11
	RR	3	3

*, unmatched results of the individual sample at position 190 SNP, ^#^, unmatched results of the individual sample at position 215 SNP. For the following SNPs/mutations: position 190: SS (c.190CC), RR (c.190AA or c.190AG), RS (c.190CA or c.190CG), position 215: SS (c.215GG), RR (c.215TT), RS (c.215GT), and position 2134: SS (c.2134TT), RR (c.2134CC), RS (c.2134TC).

#### Resistance allele frequencies in *Rhipicephalus linnaei* populations in Thailand

**Fig. 3:**
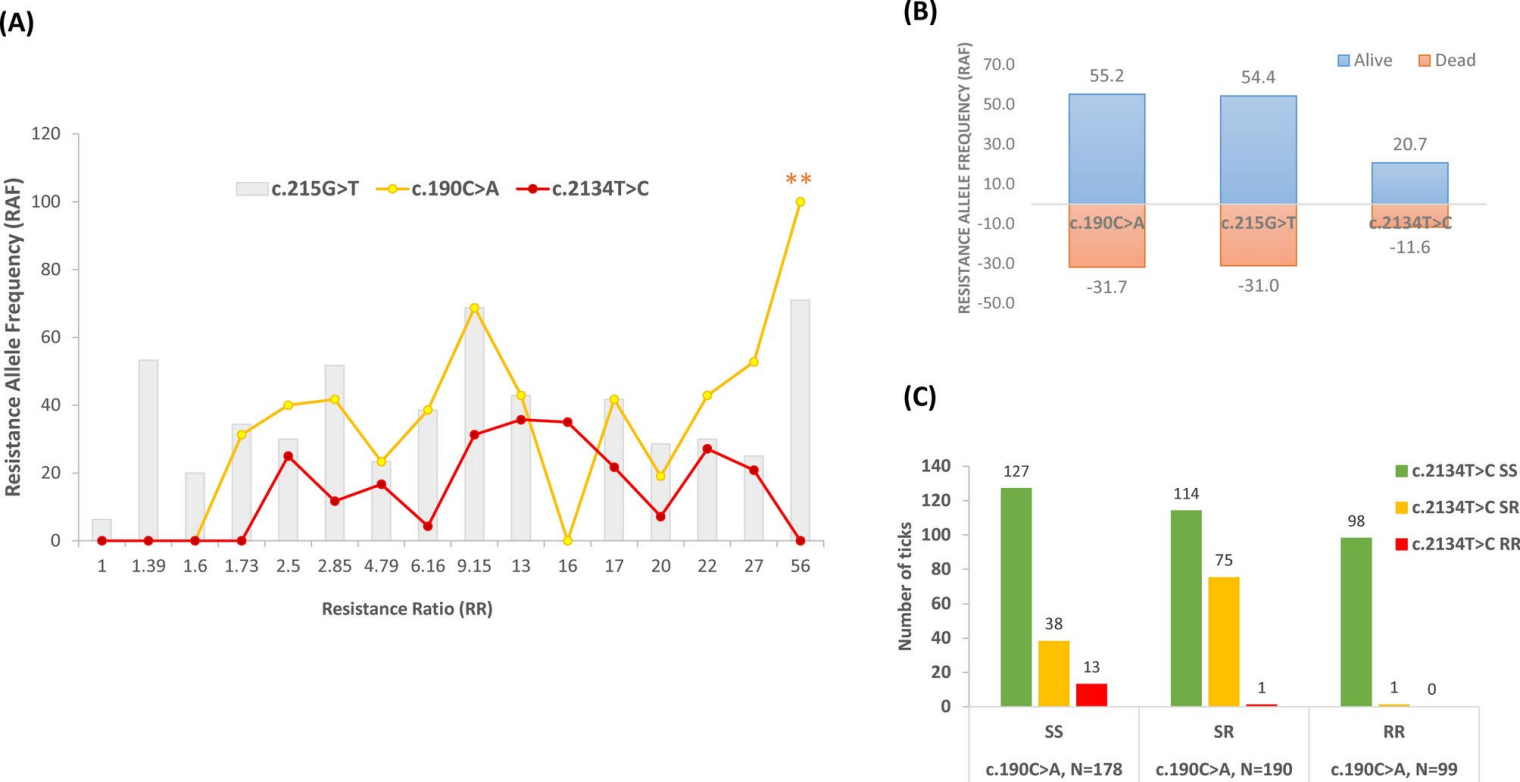
Allele distribution of three loci (c.190C > A, c.215G > T, and c.2134T > C) of the *vgsc* gene. (**A**), the distribution of three SNPs according to Resistance Ratio (N = 379). (**), new allele was detected in this population (c.190AG) from Bangkok (Phasichareon) with RR = 56.00. (**B**), Resistance allele frequency (RAF) of three SNPs between dead and alive populations. (**C**), Co-occurrence of SNPs at positions 190 and 2134 genotypes (SNP at position 190; SS: c.190CC, SR: c.190CA & c.190CG, RR: c.190AA & c.190AG, and SNP at position 2134; SS: c.2134TT, SR: c.2134TC, RR: c.2134CC).

Table 1 presents the resistance allele frequency (RAF) for SNPs c.190C > A, c.215G > T, and c.2134 T > C, accompanied by the RR values and a 95% confidence interval, from larvae obtained from engorged female ticks collected from locations across Thailand. Resistance allele frequency values for c.190C > A and c.2134T > C in populations with low resistance levels (1.00–1.73) range from 0 to 31.30, indicating that most ticks in these locations carried susceptible genotypes for these loci. In contrast, the RAF value for c.215G > T ranges from 6.25 to 53.3 at the same resistance level and remains relatively constant despite increasing resistance levels (Table 1 and Fig. 3A). In tick populations with the RR starting at 2.50, RAF values for c.190C > A begin to appear at 40.00 and remain at relatively similar frequencies as RR increases, except for ticks from Krabi. The RAF values for c.2134T > C starts to emerge at the RR value > 2.50 and remains constant as the resistance level increases (Fig. 3A).

Among highly resistant populations, single mutation at either positions 190 or 2134 were detected in tick populations, such as those from Krabi (c.2134T > C, RAF = 35.0) and Phasichareon, Bangkok (c.190C > A RAF = 100) with new allele (c.190AG) was detected in both engorged female and larvae (Supplementary Table S5 online). Additionally, new alleles c.190CG and c.190AG were identified in larvae obtained from female *Rh. linnaei* ticks collected from two locations in Chonburi provinces, both exhibiting high permethrin resistance. Specific genotypes of these two locations are as follows; Bang Lamung (N = 30) had 8 c.190AA (p.Ile64Ile), 2 c.190CG (Leu64Val), 13 c.190CA (Leu64Ile), and 7 c.190CC (Leu64Leu); Ban Chang (N = 33) had 21 c.190AA (Ile64Ile), 1 c.190CG (Leu64Val), 10 c.190AG (Ile64Val), and 1 c.190CA (Leu64Ile).

Resistance allele frequency of three SNPs detected in the genotyped ticks were plotted according to their resistance levels on the X-axis (Fig. 3A), and by the living and dead populations derived from the bioassay (Fig. 3B). The distribution of the resistance allele frequency (RAF) of c.215G > T appeared with no specific pattern according to resistance level; however, RAF values for both c.190C > A/c.190C > G and c.2134T > C increased as the resistance level increased (Fig. 3A). There are two populations with zero RAF value for c.190C > A (at RR 16.00) and c.2134T > C (at RR 56.00); however, this was compensated for with high resistance allele frequency of c.2134T > C (RAF = 35.00) and with new alleles c.190C > A and c.190C > G (RAF = 100), respectively. A similar observation was found in the population from Chonburi province (Ban Chang, Table 1) with a strongly resistant phenotype where the SNP at position 190 had a high RAF (90.90) but c.2134 T > C had an RAF of 3.0. The resistance allele frequency between the living and dead populations from the LPT was evaluated and it was found that RAF values are higher in the living population than the dead in all SNPs studied (Fig. 3B). Comparing the proportion of ticks carrying resistant (RS, RR) genotypes in susceptible (SS) populations for two mutations: c.190C > A/c.190C > G and c.2134 T > C (Fig. 3C), the results showed a statistically significant difference between the proportion of ticks with c.2134TC/ c.2134CC resistant genotypes (RS/RR, N = 51, RAF = 18.71%) in the c.190CC homozygous susceptible genotype (N = 178, prop = 0.257), which is lower than that of ticks with c.190CA/c.190CG/c.190AA/c.190AG resistant genotypes (RS/RR, N = 212, RAF = 45.72%) in the c.2134TT homozygous susceptible genotype (N = 339, prop = 0.528) with Chi-square = 30.264, df = 1, *p*-value = 3.771e-08. This result indicates that the c.190C > A/c.190C > G mutation is more prevalent than c.2134T > C in permethrin-resistant *Rh. linnaei* populations in Thailand. When testing the association between these two mutations of susceptible and resistant genotypes using a 2-by-2 contingency table, the Pearson’s Chi-squared test with Yates’ continuity correction revealed that there is no association between the two (Chi-square = 0.134, df = 1, *p*-value = 0.712).

### Correlation between phenotype and genotype in *Rhipicephalus linnaei* ticks in Thailand

**Fig. 4:**
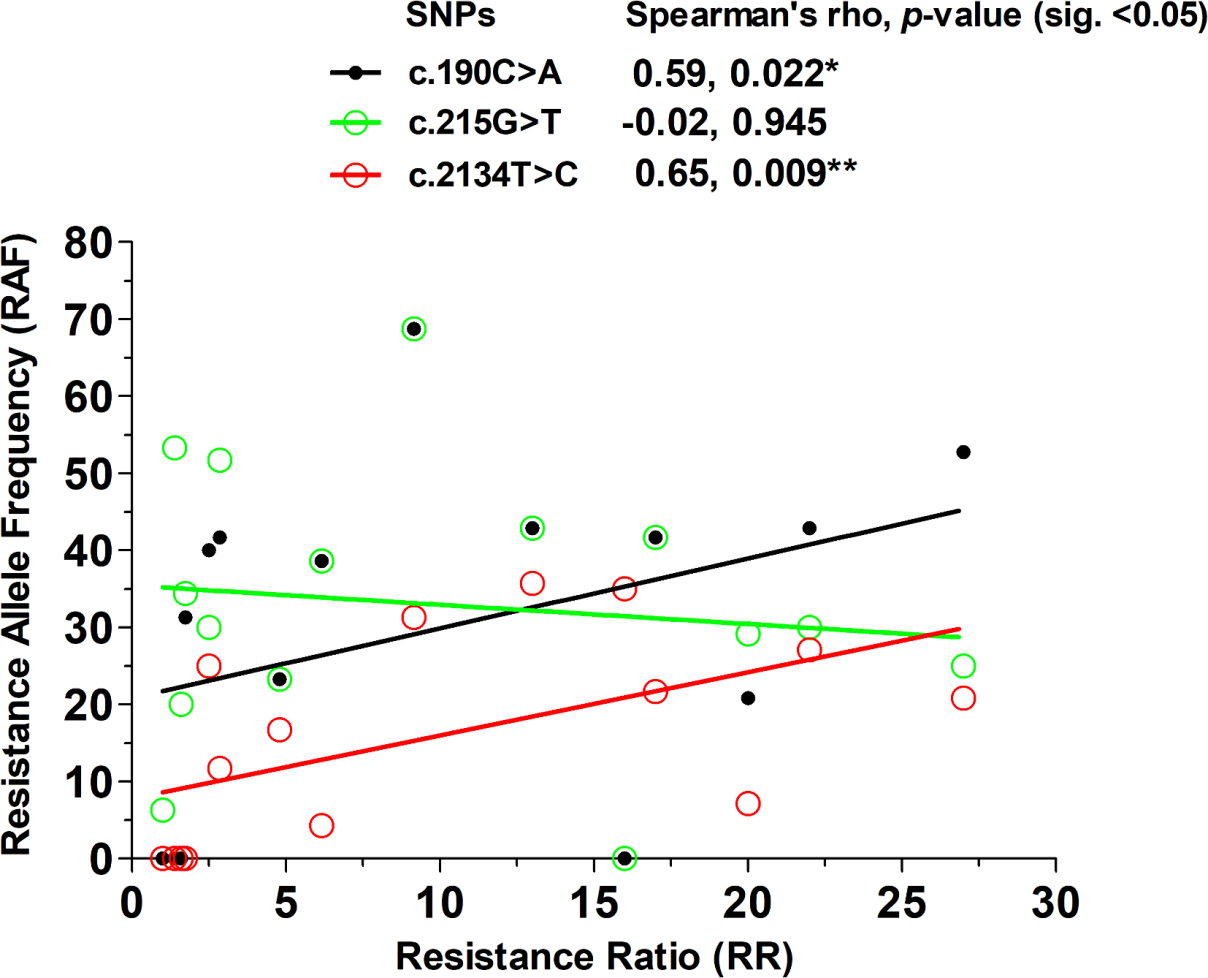
Correlation graph between Resistance Ratio (RR) from the bioassay test and the resistance allele frequency (RAF) of three loci of the *vgsc* gene. The correlation test was performed with Spearman’s rank correlation rho in RStudio, version 2024.9.0.375, package “corrplot”.

Correlation analysis was performed to determine the association between the occurrence of SNPs, as the resistance allele frequency (RAF), and the resistant phenotypes (RR values). In this analysis, the RR value was a measure of the resistance level to permethrin and served as the dependent variable. The number of resistant alleles (RR and RS) at each position was used to calculate the resistance allele frequency in the tick population and served as the independent variable. The analysis was conducted for c.190C > A, c.215G > T, and c.2134 T > C SNPs. However, this analysis excluded the tick population from Bangkok-2 (Phasichareon) since it contained a mutation of c.190AG which was different from the rest of the tick populations in the analysis. Genotypes of larvae from Chonburi were also not included, as permethrin resistance in this population was so high we were unable to calculate the true RR. Mortalities of 0.9% and 12.0% were observed in larvae from these two locations after exposure to 10% (w/v) of permethrin, the highest concentration used in this study. The results showed a significant correlation between RAF and RR values for the c.190C > A mutation (Spearman’s rho coefficient t = 0.59, *p*-value = 0.022) and c.2134T > C (Spearman’s rho coefficient = 0.65, *p*-value = 0.009) but not for the c.215G > T mutation (Spearman’s rho coefficient = − 0.02 *p*-value = 0.945) (Fig. 4). This result indicates that both the c.190C > A and c.2134T > C mutations are associated with the resistant phenotype in ticks, with an increase in resistance allele frequency observed in populations with higher resistance levels. A correlation test for c.170T > C and c.2166T > C revealed no correlation between its RAF and corresponding resistance ratio values (spearman’s rho = 0.040, − 0.362, *p*-value = 0.893, 0.225, respectively) (Supplementary Fig. S6 online). Due to the limited c.2166T > C sequence data, the results are not definitive.

#### Haplotype identification and statistical analyses

Eighteen haplotypes (Hap001-Hap018) were identified from genotyping data (three mutations at positions 190, 215, and 2134) of *Rh. linnaei* populations in Thailand, including only one wild-type or susceptible genotype, Hap002 (nt: CGT, aa: L-G-F), which has the second highest frequency (84/457, 18.4%) after Hap001 (nt: MKT, aa: L/I-G/V-F), containing 87 individuals (19.0%) (Table 3). The majority of the haplotypes (13/18 haplotypes) identified in this study contain heterozygous genotypes belonging to one of the three mutations (positions 190, 215, and 2134) in the *vgsc* gene. Six haplotypes contain fewer than five individuals each, with frequency ranges from 0.2 to 0.7%. The new alleles identified in this study belong to Hap006 (nt: RKT, aa: I/V-G/V-F), with 28 individuals in this group (6.1%), and Hap015 (nt: SGY, aa: L/V-G-F/L), with only 3 individuals in the group (0.7%).

**Table 3:**
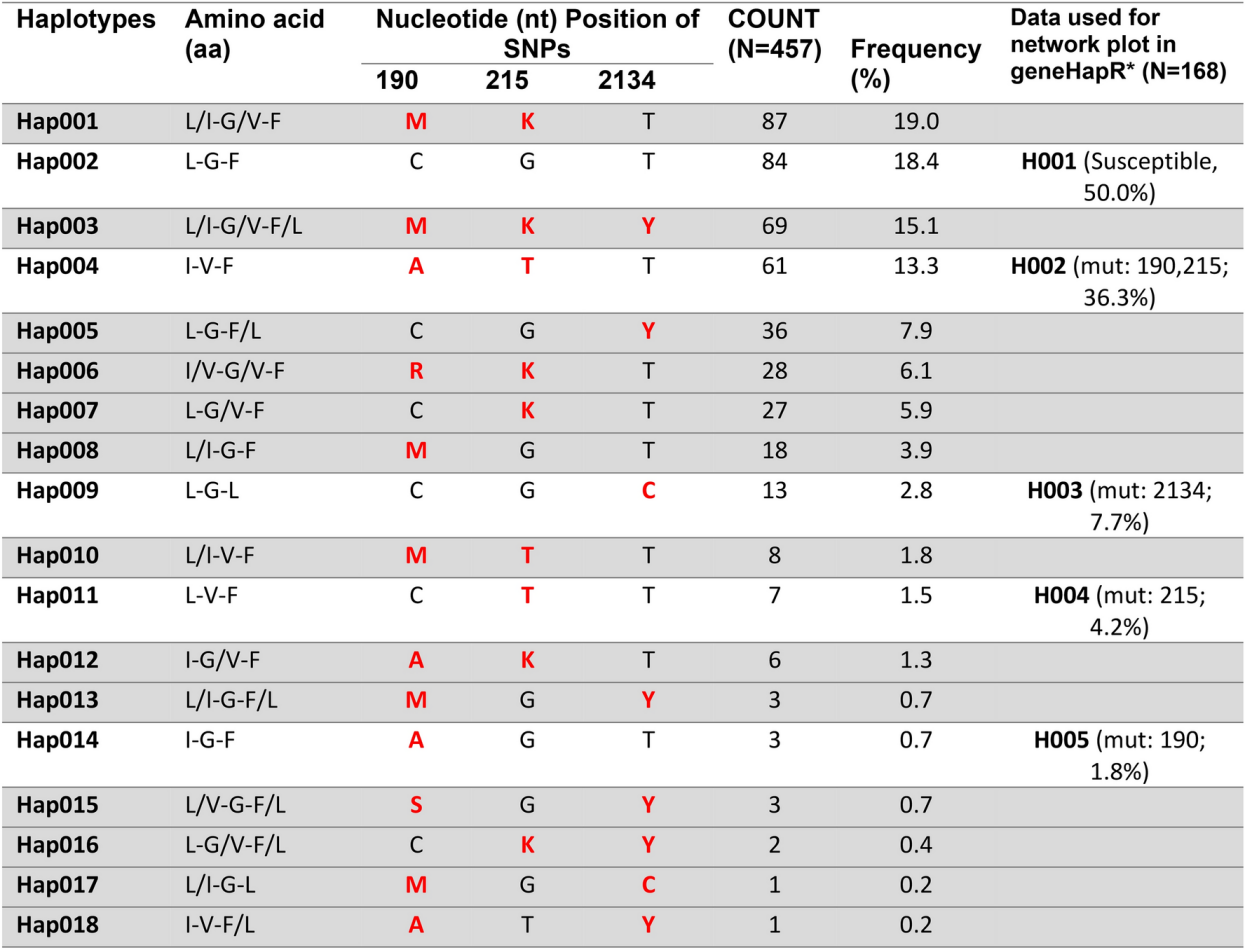
Haplotypes of permethrin-resistant *Rh. linnaei* ticks identified in this study. 457 individuals were included in the haplotype analysis.

(*), Haplotypes identified by the geneHapR software, which is limited to homozygous genotypes, all heterozygous and missing data were removed before analysis, including the step of network plotting. Highlights in grey color denote haplotypes containing heterozygous genotype(s). Red letters represent mutations at each position. H001-H005 are haplotypes identified by the geneHapR package, with their genotypes and % frequency in parentheses. IUPAC nucleotide code: M = A or C, K = G or T, Y = T or C, R = A or G, S = C or G. Data (positions 190, 215, and 2134) used in this analysis were genotyped by DNA sequences and PCR-RFLP assay. IUPAC amino acid code: G = Glycine, F = Phenylalanine, I = Isoleucine, L = Leucine, V = Valine.

**Fig. 5:**
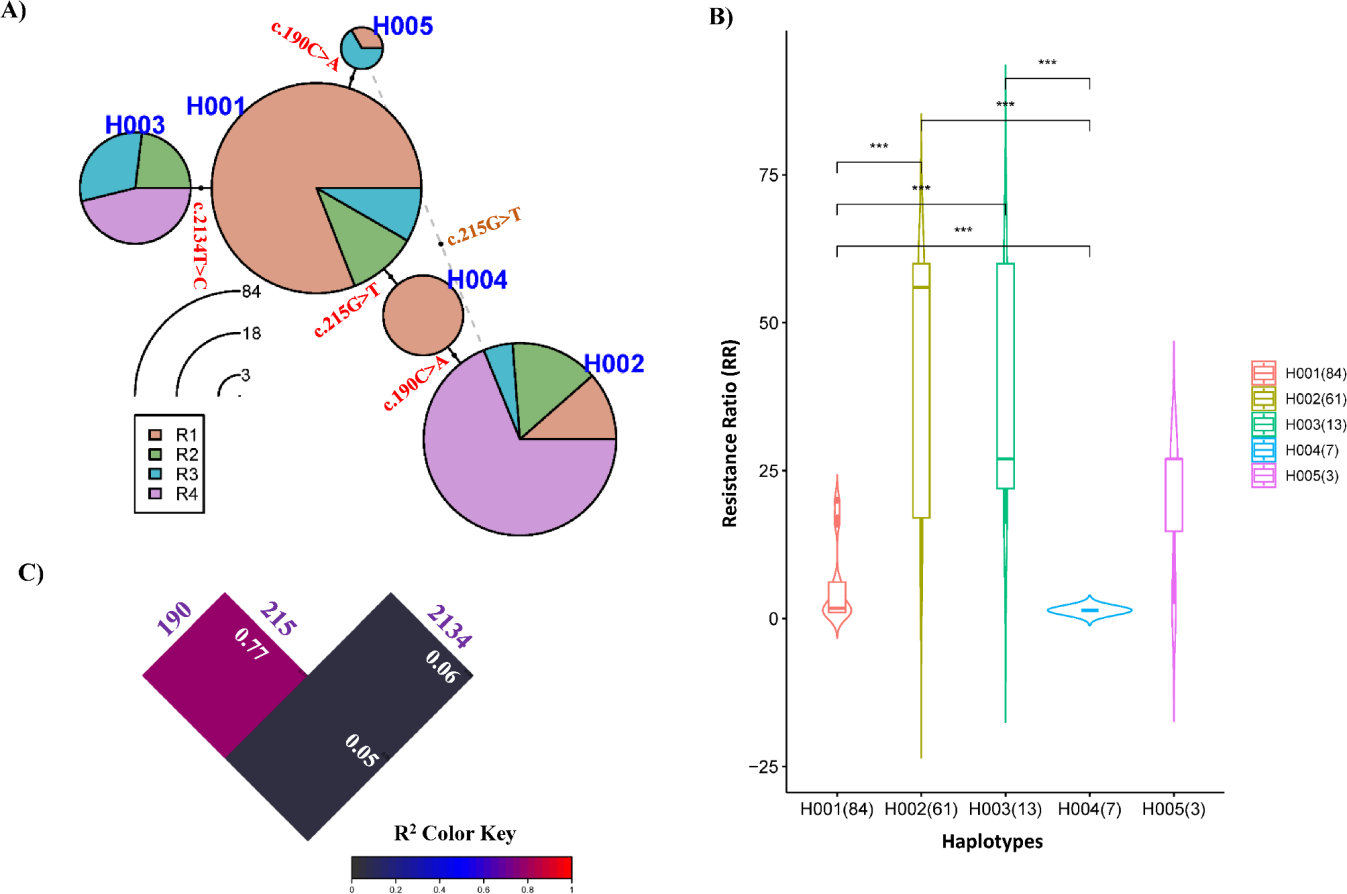
A haplotype network for H001-H005 (with heterozygous genotypes removed) represents the evolutionary relationships between haplotypes (**A**). Variants between haplotypes are represented as a dot with SNP name in red letters. Circle sizes are plotted according to the frequency of each haplotype, and the pie angles are proportionate to the number of individuals in each resistance range (resistance ratio ranges = R1:1–9, R2:10–19, R3:20–29, and R4: ≥ 30). Their pairwise comparison of resistance levels between haplotypes (**B**) with *p*-values (*** < 0.001, * < 0.05) are indicated on top of the lines. The linkage disequilibrium plot (‘plot LD heatmap’ function) among three mutations (positions 190, 215, and 2134) is shown (**C**). R^2^ values and mutation positions are indicated in each diagonal and at the top of each one, respectively. All analyses and visualizations shown in this Figure were performed using the “geneHapR” package in RStudio, version 2024.9.0.375.

The same data set was subjected to haplotypic statistical analysis using the geneHapR package in RStudio, version 2024.9.0.375. Due to software limitations, heterozygous genotypes and missing data were eliminated by default (289 individuals were removed). The software identified five haplotypes (H001-H005) (Table 3). The H001 haplotype contains only susceptible genotypes (84/168, 50%) for all three mutations, H002 (61/168, 36.3%) has two mutations (c.190C > A and c.215G > T), and H003-H005 each contains only one mutation: c.2134T > C, c.215G > T, and c.190C > A (13/168 (7.7%), 7/168 (4.2%), 3/168 (1.8%), respectively). The network plot (Fig. 5A) showed the possible relationships between haplotypes. The H001 haplotype contains the majority of individuals tested (84/168), with most of them belonging to R1 (RR = 1–9) and smaller proportions in R2 (10–19) and R3 (RR = 20–29), respectively. From the H001 haplotype, ticks could evolve into three haplotypes by one mutational step at positions c.190C > A, c.215G > T, and c.2134T > C, evolving into H005, H004, and H003, respectively. The H002 haplotype has one additional mutation from H004 (c.190C > A) or, alternatively, it could come from H005 (c.190C > A) with an additional mutation at c.215G > T. These ticks became more resistant to permethrin since the majority of ticks (42/61, 68.9%) in H002 belong to R4 (RR ≥ 30). While H003 has only one mutation at c.2134T > C from H001, almost half of the ticks were highly resistant to permethrin (ticks in R4). Phenotypic association between haplotypes and resistance levels was analyzed (Fig. 5B) and revealed that ticks carrying haplotypes H002 or H003 were significantly more resistant to permethrin than other haplotypes, with *p*-value < 0.001. The linkage disequilibrium analysis (Fig. 5C) revealed that the SNPs at positions 190 and 215 were closely linked (R^2^ = 0.77) compared to other pairs of SNPs (positions 190–2134 or positions 215–2134). From this evolutionary relationship analysis, the data revealed that the point mutation at c.2134T > C in Domain III of the *vgsc* gene in ticks results in strong resistance to permethrin. A c.190C > A mutation alone may confer some resistance, but it requires an additional mutation at c.215G > T to reach the same level of resistance observed in ticks carrying the point mutation at c.2134T > C.

### *Rhipicephaluslinnaei* in Thailand

Molecular identification was conducted on 3–4 larvae from each location using mitochondrial genes; cytochrome oxidase subunit I (*COX*I), 12S rDNA, and 16S rDNA. The results show high sequence similarity among *Rh. linnaei* samples collected throughout Thailand with 99.6%-100% identity for *COX*I and 12S rDNA genes, and 100% for 16S rDNA sequences (see Supplementary Fig. S8-S10 online). For 12S rDNA sequences among Thai populations, there are 3 sequences from Petchabun (PNB) that have 99.6% identity with the others, with only one nucleotide change detected. All Thai sequences of *COX*I, 12S rDNA, and 16S rDNA fell into the *Rh. linnaei* tropical lineage clade along with *Rh. linnaei* from Laos, Fiji, and Australia, with sequence identity ranging from 99.6 to 100%. Given this high sequence similarity among Thai *Rh. linnaei* populations, there is no evidence of genetic structure in Thai tick populations. The population genetic analysis of *Rh. saguineus* s.l. OSU-USA laboratory population (Pop 1) in comparison to Thai populations (Pop 2) was performed using the STRUCTURE software with *COX*I sequences (N = 64). The data showed that while there is no genetic structure among Thai *Rh. linnaei* populations, there is a clear differentiation between USA laboratory and Thai populations (deltaK = 2), with 100% probability for each tested individual being grouped into the respective inferred clusters (see Supplementary Figure S11).

## Discussion

This study is the first, to our knowledge, to perform a cross-sectional survey of permethrin resistance in larvae obtained from engorged female *Rh. linnaei* populations in Thailand. The data revealed resistant phenotypes across the country of resistance levels varying from province to province (RR ranges = 1.00–56.00, median = 7.66, mean = 12.64). A comprehensive study comparing the permethrin-resistant phenotypes using the FAO LPT bioassay^46^ with their genotypes was performed. This study revealed a significant correlation between the resistant phenotype and mutations at c.190C > A in Domain II and c.2134T > C in Domain III of the voltage-gated sodium channel gene. Previous studies reported resistance of this tick to ivermectin and fipronil in Thailand^30,31^. However, these studies were conducted in a limited number of locations, and tick genotyping was not reported.

The mutations (c.170T > C, c.190C > A, c.215G > T, and c.2134T > C) previously observed in *Rh. microplus* were also detected in this study. These mutations have also been reported in other countries, and c.2134T > C and c.2134T>A have been recognized as important mutations correlated with permethrin resistance in *Rh. sanguineus* and *Rh. microplus*^11,21,26,47^, respectively. Similar to pyrethyroid-resistant *Rh. microplus,* which displays a c.2134T > A mutation (nt: Thymine to Adenosine, aa: Phe to Ile) on Domain III segment VI of the *vgsc* gene^29,46,47^, *Rh. sanguineus* also exhibits a mutation at the same location. However, in *Rh. sanguineus*, the substitution is cytosine (nt: c.2134T>C, aa: Phe -> Leu), as shown by Klafke et al.^48^ and in this study. The c.2134T>C mutation has been detected mainly in the tropical linage of *Rh. sanguineus* (recently reclassified as *Rh. linnaei*) rather than the temperate linage^21,26^. This study also highlights the importance of the mutation in Domain II (position 190) and new alleles (c.190CG and c.190AG) that were observed in populations with a highly resistant phenotype (Bangkok-2 and two locations in Chonburi). The mutation c.190C > G was previously observed in *Rh. microplus* by Stone et al.^48^, but the c.190AG allele has not been previously observed. It is important to note that for some regions, the majority of larvae (24,697/33,095, 74.6%) used in the study were primarily derived from a single egg mass. To draw reliable conclusions about rarer alleles, a larger sample size from all locations is needed to determine their frequency accurately. Future work will include phenotypic and genotypic analysis of a more diverse sample of larvae from a larger number of females, in addition to genotypic analysis of other life stages.

In this study, high permethrin resistance was observed in two locations in Chonburi, where LC-50 could not be determined because only 0.9–12% mortality was observed at 10% (w/v) of permethrin. In these tick populations and ticks from Bangkok (Phasichareon) with RR = 56.00, new alleles c.190AG and c.190CG were detected at position 190 (Domain II), changing the amino acid at this position from Leucine (Leu) to Valine (Val) or to Isoleucine (Ile) and Valine (Val). Although they are all hydrophobic amino acids, the 3D structure of the protein affected by the substitution of these new alleles should be studied further. Generally, both mutations (positions 190 and 2134) were detected in moderately to highly resistant ticks. However, in some populations with low and high resistant phenotypes, a single mutation occurred either at positions 190 or 2134. For example, no or low frequency of the c.2134T > C mutation (RAF = 0–3.0) was detected in Bangkok (Phasichareon, N = 31) and Chonburi (Ban Chang, N = 33), respectively. These two tick populations carried c.190AA and c.190AG alleles with high resistance allele frequency ranging from 90.9 to 100%. A similar observation was made in ticks from Krabi (N = 10), where only the c.2134T > C mutation was detected (RAF = 35) and no mutation at positions 190 and 215 were detected. However since the sample size was small, a definitive conclusion cannot be drawn at this time. Most mutations detected are nonsynonymous, where the substitutions occur at the first or second position of the translation codon, leading to changes in the amino acid. The correlation test between c.190C > A and c.2134T > C mutations with resistance phenotypes indicates that as the resistance allele frequency increases, so does the phenotypic resistance level of the populations. This confirms the significant association between phenotypic resistance and the mutations detected in the *vgsc* gene reported in this study. Some additional synonymous mutations (no amino acid change) were also observed; c.2024T > C, c.2033C > T, and c.2048G > A^49^, and c.2166T > C (in this study). However, their roles in the resistance mechanism are unknown and require further study.

Haplotype analysis found that the majority of ticks (N=84, 50.0%) have susceptible genotypes for all three main mutations (H001), while the combination of mutations (positions 190 + 215, H002) is the major resistant genotype (36.3%) observed, followed by H003 with only c.2134T > C mutation (7.7%), H004 (G215T, 4.4%), and H005 with only a point mutation at c.190C > A (1.8%). According to the evolutionary relationships depicted in the network plot by resistance level, the important point mutation conferring permethrin resistance is still at c.2134T > C; however, ticks with two mutations at positions 190 and 215 can strongly resist permethrin at the same level as ticks with a single mutation at c.2134T > C. Moreover, both positions 190 and 215 variants are closely linked and generally detected simultaneously in resistant ticks. A haplotype network analysis in this study illustrated the potential combination of SNPs or alleles (in two DNA fragments of the *vgsc* gene) originating from a single parent, which might be linked to resistant phenotypes. This analysis does not account for selection pressure that can drive the development of different pathways.

Permethrin resistance mechanisms in *Rh. sanguineus* and *Rh. linnaei* do not rely solely on mutations at the sodium channel, the target gene in this study. Instead, resistance may also result from elevated metabolic detoxification enzymes such as cytochrome P450, esterase, or glutathione-*S*-transferase or involve a complex of several metabolic processes^15,23^. Several studies have also reported that both mechanisms (mutation and metabolic detoxification) play an important role in permethrin resistance in *Rh. microplus* ticks^28^. Therefore, the mutations reported in this study might be only one aspect of permethrin resistance in *Rh. linnaei* in Thai populations, and further investigation should be carried out to determine if other mechanisms play a role in this process.

Monitoring acaricide resistance in tick populations can help guide tick control programs to effectively mitigate the risk of disease transmission to both animals and humans. The PCR-RFLP assay developed in this study could be a useful tool to survey *Rh. linnaei* populations in other regions in Thailand and in Southeast Asia. This assay could help evaluate the status of permethrin resistance and the impact of target site resistance in *Rh. linnaei* in the region. The tick populations evaluated in this study were limited to Thailand and only the tropical lineage was detected. Future work will include populations from additional locations in Southeast Asia and the Pacific, encompassing the temperate lineage and varying patterns of permethrin exposure.

### Disclaimer

Material has been reviewed by the Walter Reed Army Institute of Research. There is no objection to its presentation and/or publication. The opinions or assertions contained herein are the private views of the author, and are not to be construed as official, or as reflecting true views of the Department of the Army or the Department of Defense. Research was conducted under an IACUC-approved animal use protocol in an AAALAC International—accredited facility with a Public Health Services Animal Welfare Assurance and in compliance with the Animal Welfare Act and other federal statutes and regulations relating to laboratory animals.

## Methods

### Ticks

Field populations: Engorged female ticks of the species *Rhipicephalus linnaei* were collected from domestic canines living in and around households within villages across Thailand from July 2022-July 2024, as detailed in Table 1. Owners and local residents assisted in collecting ticks from domestic animals following IACUC-approved guidance from study personnel. Engorged ticks were individually placed in vials for transportation to the laboratory for rearing. Ticks were morphologically identified using taxonomic keys^50^. The collection was performed in adherence to a WRAIR-AFRIMS IACUC-approved protocol. The engorged females, along with egg masses and larvae, were housed within the incubation chamber, which followed a 12-h day and 12-h night cycle, and maintained a temperature of 25 ± 2 °C and a humidity level of 80–90%. A plastic acrylic box with an adjustable lid was used, containing a glass of water (200 ml) to keep the humidity around 80–90%, monitored by a hygrothermograph. Engorged ticks laid their eggs in vials, and eggs hatched into larvae within a period of 24–27 days under the laboratory conditions described above. Larvae aged between 14 to 18 days were used for the bioassay.

*Rhipicephalus sanguineus* s.l. (as comparison population) was purchased from the Tick Rearing Facility at Oklahoma State University (OSU), USA. The tick colonies were maintained at the insectary facility in the Entomology Department at WRAIR-AFRIMS. International Cancer Research (ICR) mice (*Mus musculus*) from a Charles River Technology (Bio-LASCO, Taiwan) colony maintained by the Department of Veterinary Medicine, WRAIR-AFRIMS were utilized as the blood source for all tick life stages. The entire feeding procedure, along with all necessary safety precautions, was reviewed and approved by the WRAIR-AFRIMS IACUC committee (PN# 21-13).

### Bioassay (larval packet test)

A dose–response curve was established for permethrin, applicable to both field-collected and laboratory-reared ticks. The bioassay was established based on the Food and Agriculture Organization’s Laval Packet Test (LPT)^51^. Technical-grade permethrin (Item: N-12848, CAS number: 52645-53-1), supplied by ChemService (West Chester, PA), was used for the study with purity of 100%. Permethrin was dissolved and diluted in trichloroethylene (TCE) and olive oil in a 2:1 volume ratio. Whatman No.1 filter papers were treated with sequentially diluted permethrin (4–5 dilutions). Filter papers were then left to dry under a fume hood for a period of two hours. Roughly 80–100 larval ticks obtained by rearing engorged female ticks collected from locations across Thailand, aged between 14 and 18 days post-eclosion, were used for each active ingredient concentration and placed in a filter packet. These packets were stored within an environmentally controlled container (25 ± 2 °C, 80 ± 10% Relative Humidity). A total of 2–4 packets were prepared per chemical concentration with 4–5 permethrin concentrations tested per population, along with a solvent-only treated filter paper as a control. The concentrations used in the LPT assay ranged from 0.10% to 10.0% (w/v) or 0.015 mg/cm^2^ to 1.57 mg/cm^2^ (Army uniform treatment: 0.095–0.135 mg/cm^2^). Mortality was assessed after 24 h, with the counts of both live and dead ticks being recorded under a stereomicroscope with magnification ranging from 10 × to 50x (Supplementary Fig. S1 online). Ticks were classified as dead if they did not respond to a probe by a paintbrush, to the CO_2_ from human breath, or were unable to walk^22^. Ticks that showed leg movement but could not walk were also categorized as dead. Dead and surviving ticks were separated and pooled by permethrin concentration into separate tubes and stored at − 80 °C in ethanol for DNA extraction. Larvae were usually from a single engorged female, except in the following locations: Nakhonprathom, Lopburi, and Suratthani, where larvae were obtained from two engorged females. In Chantaburi province, larvae were a mixture from five engorged females collected from the same village.

### Probit analysis

Lethal concentration values (LC-50, LC-90, and LC-95) and 95% confidence intervals were determined for each tick species using Probit analysis (package “BioRassay” in R programing language, RStuio version 2024.9.0.375)^51^ which generated the probability curve. Abbott’s correction from control mortality (if > 5% mortality) was applied to the data set^52,53^. Resistance ratios (RRs) at the LC-50 value were determined for field-collected tick species using the least susceptible local population as a baseline (Lamphun province). The similarity of mortality-dose regression among different populations was tested using the Likelihood Ratio Test (LRT) in BioRassay package in RStudio, version 2024.9.0.375. This test computed pairwise comparisons of each pair of isolates, followed by sequential Bonferroni correction (see Supplementary Fig. S2 online).

### PCR amplification, DNA sequencing and phylogenetic tree analysis

DNA was isolated from individual ticks (larva and engorged female) using a previously published protocol (Supplementary Table S7 online)^54^. Two target regions of the voltage-sensitive sodium channel gene; 1) domain II S4-5 linker region, 2) region in S6 domain III were amplified using primers and PCR conditions following the previous published protocols (Supplementary Fig. S3 online)^21,49^. Briefly, 20-µL reaction consists of 1.0 U iProof™ High-Fidelity DNA polymerase (Bio-Rad, Hercules, CA), 200 µM dNTPs, MgCl_2_ and primer concentrations as indicated (see Supplementary Fig. S4 and Table S2 online). The PCR cycle started with 98 °C for 30 s, followed by 35 cycles of 98 °C, 10 s, annealing temperature for 10 s (indicated in Supplementary Table S2 online), and 72 °C for 30 s, with the final extension at 72 °C for 5 min.

Domain II S4-5 linker region was amplified by DM2F1 and DM2R1primers; however, a nested PCR was used for Domain III amplification with DM3F1 and DM3R1 primers in the first-round PCR, and then the first-round product (1:10) was used as a template in the second-round PCR amplification with DM3F2 and DM3R2 primers. Tick mitochondrial genes (12S rDNA, 16S rDNA and cytochrome oxidase subunit I) were amplified by primers obtained from previously publish protocols^55–57^. The primer sequences, annealing temperatures, and MgCl_2_ concentration in the reaction may be found in the Supplementary Table S3 online.

PCR products were cleaned by ExoSap-IT PCR product cleanup Reagent (Applied Biosystems, Foster City, CA) and sequenced using BigDye® Terminator Ready Reaction Premix and a genetic analyzer instrument and software, SegStudio™, version 1.2.4 (Applied Biosystems). Sequences were edited and assembled using DNA Sequencher® version 5.4.6 (Gene Code Corporation, Ann Arbor, MI). Sequences were aligned by Jalview—a multiple sequence alignment editor and analysis workbench, version 2.11.4.1, for graphical presentation^58^. Sequence alignment and distance matrix calculations were performed using the MUSCLE algorithm in Molecular Evolutionary Genetics Analysis (MEGA) software, version 10.0.5. A maximum likelihood (ML) phylogenetic tree was created with MEGA software, version 10.0.5 using the best fit model with 1000 bootstraps analyses for node reliability^59^.

### Restriction fragment length polymorphism (RFLP) assays

The search for restriction endonuclease (RE) enzymes that cut specific 4–6 nucleotide sequences containing SNPs and a restriction enzyme maps was performed using the BioEdit—a biological sequence alignment editor, version 7.2.5^60^. The PCR product for Domains II and III of the *vgsc* gene was cut with the RE enzyme BsaJI and MboII (New England Biolabs, Ipswich, MA), respectively. The digestion of the PCR product with the RE enzyme was done at 37 °C for 30 min. The resulting cut products were subjected to 3% gel electrophoresis, using an UltraPure™ Low-Melting Point Agarose (Invitrogen, Thermo Fisher Scientific, Carlsbad, CA), for approximately one hour. The distinctive fragment pattern for each genotype was observed by a Gel Imaging System FastGene® FAS-Nano (NIPPON Genetics EUROPE GmbH, Germany). Any samples that produced fragment patterns that could not be recognized were subjected to DNA sequencing for clarification. A restriction fragment length polymorphism (RFLP) assay was employed to evaluate a larger sample size from each population and location. During the genotyping of the remaining samples using the PCR–RFLP method, unclear RFLP patterns and randomly selected samples were sequenced to confirm the PCR–RFLP results.

### Resistance allele frequency (RAF)

$${F_{(RR,RS)}} = \left( {\left( {2{N_{RR}} + {N_{RS}}} \right)/2N\;} \right)*100$$

F is the frequency of the resistant allele. N is the total population size. This calculation provides the overall frequency of the resistant allele within the population.

### Statistical analysis

The correlation between genotypes using resistance allele frequency (RAF) values and phenotypes using Resistance Ratio (RR) values derived from Probit analysis was performed using the Spearman nonparametric method in RStudio, version 2024.9.3.375 (packages; corrplot) and GraphPad Prism, version 5.04 (GraphPad Software, Inc., Boston, MA).

The identification of haplotypes (H001-H005) and statistical analysis of the *vgsc* gene (Domain II and III) was performed using the geneHapR package in RStudio, version 2024.9.0.375^61,62^. The package also facilitated producing a haplotype network plot, phenotypic comparison, and linkage disequilibrium (LD)-block plot, which are presented in this study. For Hap001-Hap018, Microsoft Excel was used to extract unique haplotypes using “pivot table” function.

## Supplementary Information

Supplementary Information.

## Data Availability

The datasets generated during and/or analysed during the current study are available in the NCBI GenBank repository (https://www.ncbi.nlm.nih.gov/genbank/) database under the following accession numbers: PQ592534 - PQ592716 (*vgsc* Domain II), PQ592717 - PQ592899 (*vgsc* Domain III), PQ573352-PQ573369 (16S rRNA), PQ573459-PQ573478 (12S rRNA), and PQ573255-PQ573294 (*COX*I gene).
