# Supplementary material for: Mutations in the voltage-gated sodium channel associated with permethrin resistance in Rhipicephalus linnaei populations in Thailand
Source: Sci Rep. 2025 Mar 2;15:7369. doi: 10.1038/s41598-025-91600-0 (PMC11873048; doi:10.1038/s41598-025-91600-0)
Supplement: Supplementary file 1 — Supplementary Information. [file 41598_2025_91600_MOESM1_ESM.pdf]

SUPPLEMENTARY INFORMATION

1. **Larval Packet Test procedure**. A dose-response curve was established for permethrin, applicable to both field-collected and susceptible ticks. The method followed the Bioassay procedure in accordance with the Food and Agriculture Organization's Laval Packet Test (LPT) (FAO 2004).

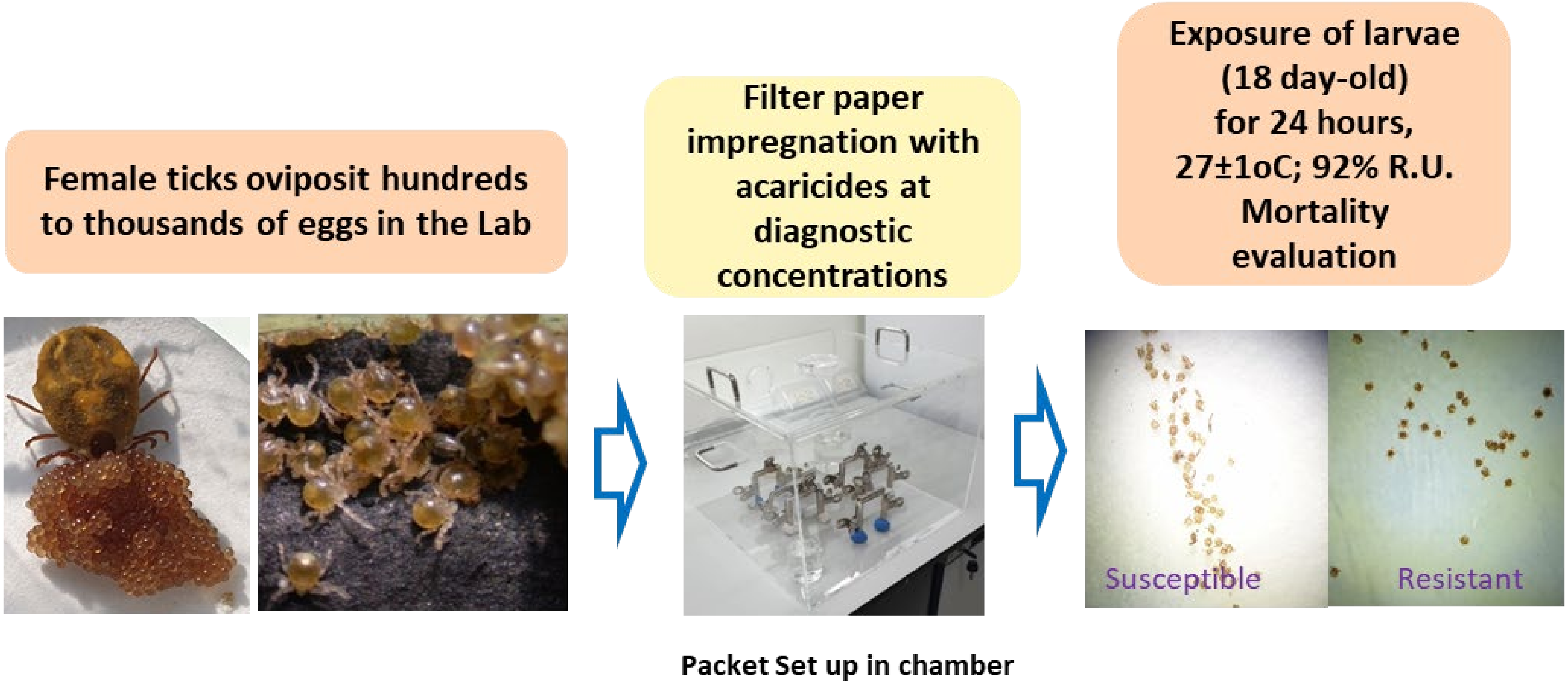

Figure S1. The diagram shows the bioassay procedure performed at the Entomology Department, WRAIR-AFRIMS.

2. **Probit analysis for the bioassay**: Lethal concentrations values (LC-50, LC-90, and LC-95) and 95% confidence intervals were determined for each tick species using Probit analysis (package "BioRassay" in R studio) which generated the probability curve and Abbott's correction from control mortality was applied to the data set. Graphs were plotted for each range of resistance: 1.00-9.15, 13.00-17.00, 20.00-56.00 (tolerate, resistance, strong resistance)

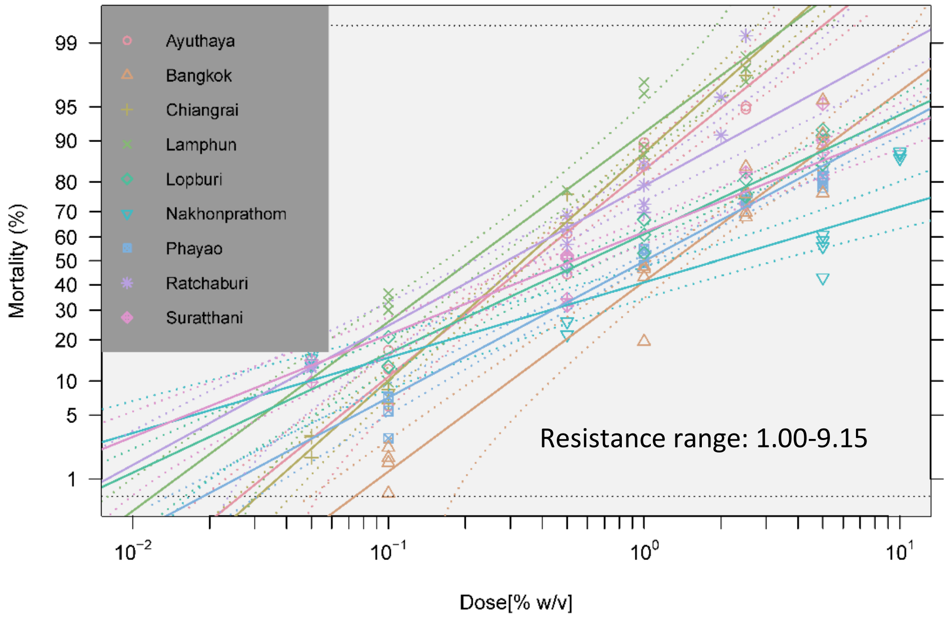

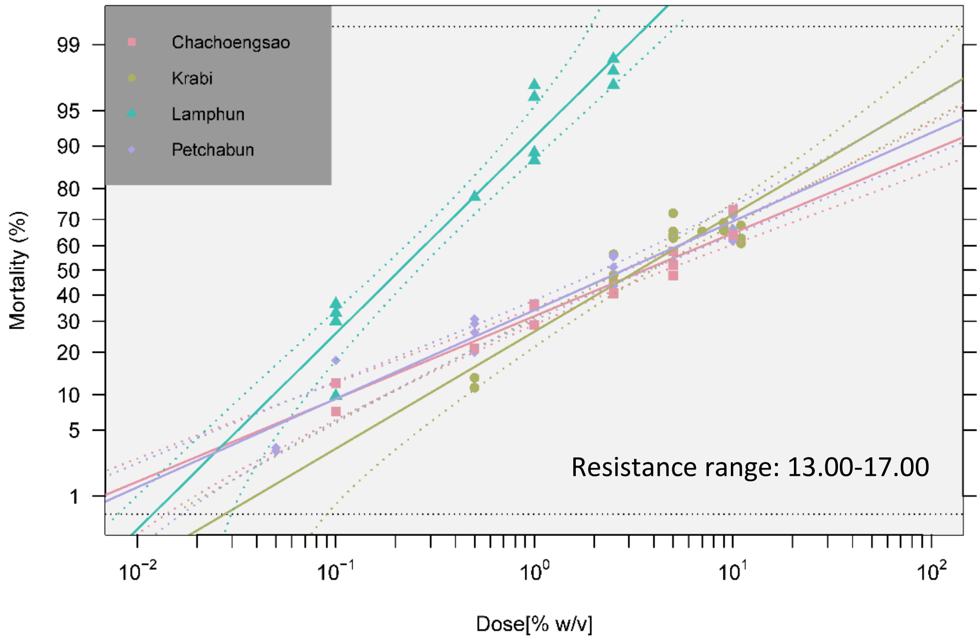

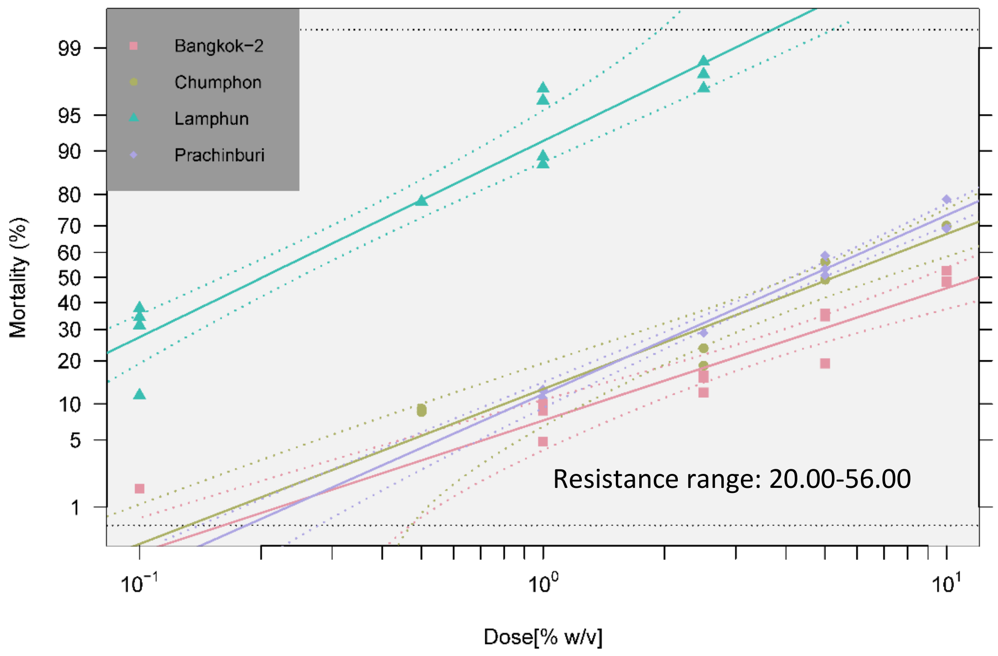

Figure S2. Probit graphs represent the resistance range of tick populations in Thailand compared to the least susceptible local population of *Rhipicephalus linnaei*.

Table S1. Toxicity of permethrin to *Rhipicephalus linnaei* populations collected from 16 provinces (18 locations) in Thailand as determined using the FAO larval packet test.

**Locations**	**GPS coordinates**	**Number of larvae (No. of replicates)**	**Slope ± SE**	**Intercept ± SE**	**Chi(p)**	***h***	***g***	**LD_{50} (min, max)**	**Resistance Ratio (95% CI)**	
									***Rh. sanguineus* as baseline**	***Rh. linnaei* (Lamphun) as baseline**
Rh. sanguineus (temperate lineage)	NA	1825 (3)	1.73±0.19	3.08±0.39	0	4.96	0.06	0.02 (0, 0.09)	1 (0.87, 1.15)	-
Ayuthaya	14.359522,100.468141	2219 (4)	2.21±0.17	0.97±0.09	0.34	2.94	0.03	0.36 (0.24, 0.49)	22 (14, 35)	1.73 (0.97, 3.08)
Bangkok (Talingchan)	13.774576,100.454502	2371 (4)	2.11±0.21	-0.16	0.07	4.63	0.04	1.29 (1.03, 1.74)	80 (17, 362)	6.16 (1.26, 30.00)
Bangkok-2 (Phasichareon)	13.70567,100.436632	1849 (3)	1.43±0.17	-1.42	0.18	2.19	0.07	12.00 (5.12, 48.00)	701 (512, 961)	56.00 (36.00, 87.00)
Chachoengsao	13.676509,101.829725	1599 (3)	0.91±0.07	-0.49	0.8	1.21	0.03	3.57 (2.48, 5.80)	237 (152, 304)	17.00 (11.00, 27.00)
Chantaburi	12.798287,102.270591	1844 (2)	1.57±0.09	-0.92	0.57	1.15	0.02	4.17 (3.15, 5.97)	251 (176, 360)	20.00 (12.00, 32.00)
Chiangrai	20.095829,99.784805	1365 (3)	2.42±0.13	1.15±0.08	0.98	1	0.01	0.33 (0.26, 0.42)	20 (12, 35)	1.60 (0.84, 3.05)
Chumphon	10.641169,99.238549	2129 (3)	1.81±0.23	-1.2	0.07	2.85	0.1	5.68 (2.58, 25.00)	342 (249, 471)	27.00 (17.00, 43.00)
Krabi	8.242495,98.907946	1724 (3)	1.21±0.11	-0.55	0.44	2.53	0.03	3.33 (2.14, 6.25)	201 (155, 261)	16.00 (11.00, 24.00)
Lamphun	18.182539,99.003476	2283 (4)	2.02±0.19	1.37±0.14	0.05	4.33	0.04	0.21 (0.10, 0.35)	13 (9, 18)	1.00 (0.62, 1.60)
Lopburi	14.982613,100.923111	2230 (5)	1.45±0.10	0.15±0.05	0.79	1.51	0.02	0.60 (0.49, 0.71)	47 (14, 91)	2.85 (1.07, 7.56)
Nakhonprathom	13.773549,100.313707	1614 (4)	0.80±0.11	-0.14	0.01	5.28	0.08	1.92 (1.10, 3.93)	116 (62, 217)	9.15 (4.52, 18.00)
Petchabun	16.572714,100.98949	1508 (4)	0.91±0.08	-0.35	0.33	1.87	0.03	2.80 (1.96, 4.43)	169 (110, 260)	13.00 (7.83, 23.00)
Phayao	19.201325,100.061173	2470 (4)	1.46±0.07	0.04	0.96	1.19	0.01	1.00 (0.89, 1.14)	61 (0.00, 2.75e+32)	4.79 (0, 4.96E+31)
Prachinburi	14.159327,101.523804	1472 (3)	2.04±0.14	-1.33	0.98	1	0.02	4.65 (3.26, 7.36)	298 (199, 395)	22.00 (14.00, 35.00)
Ratchaburi	13.507891,99.865883	1642 (4)	1.49±0.16	0.80±0.09	0.36	4.02	0.06	0.29 (0.15, 0.45)	18 (11, 26)	1.39 (0.83, 2.31)
Suratthani	9.059651,99.256014	2710 (5)	1.09±0.09	0.31±0.07	0.26	3.07	0.03	0.52 (0.36, 0.72)	32 (17, 60)	2.50 (1.23, 5.08)
Chonburi-1 (Bang Lamung)	12.899615,100.865698	1019 (2)	-	-	-	-	-	-	-	-
Chonburi-2 (Ban Chang)	12.695174,100.985838	1047 (2)	-	-	-	-	-	-	-	-

Note: Chi(p)--Chi-square goodness of fit test (p-value >0.05 indicating that the data are well fitted to the regression) and heterogeneity-related parameters (h and g), according to Finney's recommendation, which states that for a good data set, g should be smaller than 1.0 and seldom greater than 0.4., RR= Resistance Ratio, LD= Lethal Dose (% w/v). All mortality data was corrected using Abbott's formula.

Table S2. Pairwise comparison between *Rh. linnaei* populations in Thailand for their differing responses in the magnitude of the response and/or slope. The description of parameters indicated in this table was also provided.

Model test for the similarity of the mortality-dose regression for the different strains using a likelihood ratio test (LRT) and then compute the pairwise test, and corrects the model p-value using sequential Bonferroni correction. Populations that failed the linearity test were not included in this analysis (Lamphun, and Nakhon Prathom).

<u>Analysis of Deviance Table</u>

```
Model 1: mortality ~ log10(data$dose)
Model 2: mortality ~ log10(data$dose) * data$strain
  Resid. Df Resid. Dev Df Deviance  Pr(>Chi)
1       185     4563.0
2       159      361.7 26   4201.3 < 2.2e-16 ***
---
Signif. codes:  0 '***' 0.001 '**' 0.01 '*' 0.05 '.' 0.1 ' ' 1
Complete model is significant against a NULL model continuing to pair-wise comparison
```

<u>Output details</u>

```
  model.pval - significance value of ANOVA on the binomial GLM test of the strain pair
  bonferroni - significance of the model.pval with bonferroni correction
  res.Dv - residual deviance
  thr - threshold for the significance of the p-value
  str - values for the strains
  int - values for the interaction between the strain and the dose
```

	strain1	strain2	model.pval	bonferroni	res.Dv.Null	res.Dv.str	res.Dv.int	str.pval	str.thr	int.pval	int.thr
1	Ayuthaya	Bangkok	0.000	sig	1810.100	98.849	93.933	0.000	0.000	0.268	0.003
2	Ayuthaya	Bangkok-2	0.000	sig	1498.930	85.831	53.249	0.000	0.000	0.003	0.001
3	Ayuthaya	Chachoengsao	0.000	sig	1090.300	182.353	40.549	0.000	0.000	0.000	0.001
4	Ayuthaya	Chantaburi	0.000	sig	1568.420	63.916	40.854	0.000	0.000	0.003	0.001
5	Ayuthaya	Chiangrai	0.377	non-sig	NA	NA	NA	NA	NA	NA	NA
6	Ayuthaya	Chumphon	0.000	sig	1154.920	51.839	46.485	0.000	0.000	0.194	0.002
7	Ayuthaya	Krabi	0.000	sig	1213.590	150.369	80.050	0.000	0.000	0.000	0.001
8	Ayuthaya	Lopburi	0.000	sig	1051.000	97.753	40.945	0.000	0.000	0.000	0.001
9	Ayuthaya	Petchabun	0.000	sig	1237.250	192.466	55.575	0.000	0.000	0.000	0.001
10	Ayuthaya	Phayao	0.000	sig	1516.070	88.579	46.066	0.000	0.000	0.000	0.001
11	Ayuthaya	Prachinburi	0.000	sig	1087.320	37.669	33.763	0.000	0.000	0.179	0.002
12	Ayuthaya	Ratchaburi	0.008	non-sig	1189.270	109.282	77.573	0.743	0.008	0.005	0.001
13	Ayuthaya	Suratthani	0.000	sig	1302.300	176.637	69.274	0.000	0.000	0.000	0.001
14	Bangkok	Bangkok-2	0.000	sig	1614.620	105.154	88.432	0.000	0.000	0.045	0.001
15	Bangkok	Chachoengsao	0.000	sig	1309.730	190.797	75.732	0.000	0.000	0.000	0.001
16	Bangkok	Chantaburi	0.000	sig	1702.200	84.437	76.037	0.000	0.000	0.112	0.002
17	Bangkok	Chiangrai	0.000	sig	1658.760	82.349	71.078	0.000	0.000	0.064	0.002
18	Bangkok	Chumphon	0.000	sig	1375.860	82.096	81.667	0.000	0.000	0.746	0.010
19	Bangkok	Krabi	0.000	sig	1504.200	160.661	115.233	0.000	0.000	0.001	0.001
20	Bangkok	Lopburi	0.000	sig	1430.610	110.047	76.128	0.001	0.001	0.003	0.001
21	Bangkok	Petchabun	0.000	sig	1448.420	201.011	90.757	0.000	0.000	0.000	0.001
22	Bangkok	Phayao	0.014	non-sig	1815.370	102.154	81.248	0.308	0.003	0.011	0.001
23	Bangkok	Prachinburi	0.000	sig	1326.030	69.003	68.946	0.000	0.000	0.895	0.025
24	Bangkok	Ratchaburi	0.000	sig	1567.910	126.761	112.756	0.000	0.000	0.075	0.002
25	Bangkok	Suratthani	0.000	sig	1621.880	184.698	104.457	0.000	0.000	0.000	0.001
26	Bangkok-2	Chachoengsao	0.000	sig	571.260	51.181	35.048	0.000	0.000	0.009	0.001
27	Bangkok-2	Chantaburi	0.000	sig	665.890	37.747	35.353	0.000	0.000	0.283	0.003
28	Bangkok-2	Chiangrai	0.000	sig	1016.710	71.914	30.394	0.000	0.000	0.000	0.001

29	Bangkok-2	Chumphon	0.001	sig	565.910	48.256	40.984	0.003	0.001	0.126	0.002
30	Bangkok-2	Krabi	0.000	sig	1094.560	75.794	74.549	0.000	0.000	0.486	0.004
31	Bangkok-2	Lopburi	0.000	sig	1094.310	35.781	35.444	0.000	0.000	0.682	0.007
32	Bangkok-2	Petchabun	0.000	sig	678.830	63.086	50.074	0.000	0.000	0.021	0.001
33	Bangkok-2	Phayao	0.000	sig	1394.340	41.255	40.565	0.000	0.000	0.544	0.005
34	Bangkok-2	Prachinburi	0.000	sig	574.630	37.962	28.262	0.000	0.000	0.036	0.001
35	Bangkok-2	Ratchaburi	0.000	sig	1232.500	73.097	72.072	0.000	0.000	0.578	0.005
36	Bangkok-2	Suratthani	0.000	sig	1182.310	68.408	63.773	0.000	0.000	0.215	0.002
37	Chachoengsao	Chantaburi	0.000	sig	706.270	68.280	22.653	0.000	0.000	0.000	0.001
38	Chachoengsao	Chiangrai	0.000	sig	843.750	163.071	17.694	0.000	0.000	0.000	0.001
39	Chachoengsao	Chumphon	0.000	sig	497.240	75.430	28.283	0.000	0.000	0.000	0.001
40	Chachoengsao	Krabi	0.024	non-sig	737.140	76.414	61.848	0.921	0.050	0.010	0.001
41	Chachoengsao	Lopburi	0.000	sig	705.710	40.957	22.744	0.000	0.000	0.001	0.001
42	Chachoengsao	Petchabun	0.405	non-sig	NA	NA	NA	NA	NA	NA	NA
43	Chachoengsao	Phayao	0.000	sig	1049.710	74.879	27.864	0.000	0.000	0.000	0.001
44	Chachoengsao	Prachinburi	0.000	sig	469.670	68.840	15.562	0.000	0.000	0.000	0.001
45	Chachoengsao	Ratchaburi	0.000	sig	842.860	99.089	59.371	0.000	0.000	0.001	0.001
46	Chachoengsao	Suratthani	0.000	sig	860.590	59.665	51.072	0.000	0.000	0.057	0.001
47	Chantaburi	Chiangrai	0.000	sig	1143.810	49.908	17.999	0.000	0.000	0.000	0.001
48	Chantaburi	Chumphon	0.004	non-sig	720.980	30.971	28.589	0.007	0.001	0.268	0.003
49	Chantaburi	Krabi	0.002	non-sig	1168.610	72.836	62.154	0.013	0.001	0.027	0.001
50	Chantaburi	Lopburi	0.000	sig	1168.620	29.584	23.049	0.000	0.000	0.028	0.001
51	Chantaburi	Petchabun	0.000	sig	819.340	78.487	37.679	0.000	0.000	0.000	0.001
52	Chantaburi	Phayao	0.000	sig	1472.720	29.292	28.170	0.000	0.000	0.337	0.004
53	Chantaburi	Prachinburi	0.011	non-sig	719.460	19.848	15.867	0.043	0.001	0.057	0.001
54	Chantaburi	Ratchaburi	0.000	sig	1306.450	60.130	59.677	0.000	0.000	0.675	0.006
55	Chantaburi	Suratthani	0.000	sig	1269.050	74.116	51.378	0.000	0.000	0.004	0.001
56	Chiangrai	Chumphon	0.000	sig	904.190	34.294	23.629	0.000	0.000	0.020	0.001
57	Chiangrai	Krabi	0.000	sig	1073.510	136.034	57.195	0.000	0.000	0.000	0.001

58	Chiangrai	Lopburi	0.000	sig	1001.700	84.019	18.090	0.000	0.000	0.000	0.001
59	Chiangrai	Petchabun	0.000	sig	976.080	173.015	32.719	0.000	0.000	0.000	0.001
60	Chiangrai	Phayao	0.000	sig	1385.340	74.994	23.210	0.000	0.000	0.000	0.001
61	Chiangrai	Prachinburi	0.000	sig	866.580	19.810	10.908	0.000	0.000	0.002	0.001
62	Chiangrai	Ratchaburi	0.000	sig	1139.050	95.698	54.718	0.882	0.017	0.000	0.001
63	Chiangrai	Suratthani	0.000	sig	1193.200	158.769	46.419	0.003	0.001	0.000	0.001
64	Chumphon	Krabi	0.000	sig	803.950	85.117	67.784	0.000	0.000	0.014	0.001
65	Chumphon	Lopburi	0.000	sig	771.040	41.648	28.679	0.000	0.000	0.016	0.001
66	Chumphon	Petchabun	0.000	sig	621.560	86.162	43.309	0.000	0.000	0.000	0.001
67	Chumphon	Phayao	0.000	sig	1116.500	39.960	33.800	0.000	0.000	0.069	0.002
68	Chumphon	Prachinburi	0.194	non-sig	NA	NA	NA	NA	NA	NA	NA
69	Chumphon	Ratchaburi	0.000	sig	908.200	69.758	65.307	0.000	0.000	0.265	0.003
70	Chumphon	Suratthani	0.000	sig	927.370	84.903	57.008	0.000	0.000	0.006	0.001
71	Krabi	Lopburi	0.000	sig	834.330	62.664	62.245	0.000	0.000	0.647	0.006
72	Krabi	Petchabun	0.034	non-sig	878.490	87.787	76.874	0.229	0.002	0.028	0.001
73	Krabi	Phayao	0.000	sig	1224.540	74.589	67.365	0.000	0.000	0.058	0.001
74	Krabi	Prachinburi	0.000	sig	NA	NA	NA	NA	NA	NA	NA
75	Krabi	Ratchaburi	0.000	sig	971.650	106.022	98.873	0.000	0.000	0.123	0.002
76	Krabi	Suratthani	0.000	sig	1029.510	92.341	90.574	0.000	0.000	0.415	0.004
77	Lopburi	Petchabun	0.000	sig	851.680	52.177	37.769	0.000	0.000	0.004	0.001
78	Lopburi	Phayao	0.000	sig	1137.560	31.719	28.260	0.000	0.000	0.096	0.002
79	Lopburi	Prachinburi	0.000	sig	705.310	32.595	15.958	0.000	0.000	0.001	0.001
80	Lopburi	Ratchaburi	0.000	sig	814.050	63.575	59.768	0.000	0.000	0.230	0.003
81	Lopburi	Suratthani	0.392	non-sig	NA	NA	NA	NA	NA	NA	NA
82	Petchabun	Phayao	0.000	sig	1190.850	84.788	42.890	0.000	0.000	0.000	0.001
83	Petchabun	Prachinburi	0.000	sig	596.770	79.498	30.588	0.000	0.000	0.000	0.001
84	Petchabun	Ratchaburi	0.000	sig	988.840	109.354	74.397	0.000	0.000	0.001	0.001
85	Petchabun	Suratthani	0.000	sig	1001.510	71.487	66.098	0.000	0.000	0.135	0.002
86	Phayao	Prachinburi	0.000	sig	1061.890	29.993	21.079	0.000	0.001	0.007	0.001

87	Phayao	Ratchaburi	0.000	sig	1274.950	64.996	64.888	0.000	0.001	0.830	0.013
88	Phayao	Suratthani	0.000	sig	1338.370	77.285	56.589	0.000	0.001	0.003	0.001
89	Prachinburi	Ratchaburi	0.000	sig	842.490	59.238	52.586	0.000	0.001	0.120	0.002
90	Prachinburi	Suratthani	0.000	sig	872.620	77.591	44.287	0.000	0.001	0.001	0.001
91	Ratchaburi	Suratthani	0.000	sig	NA	NA	NA	NA	NA	NA	NA

3. **PCR amplification, DNA sequencing and analysis**

To identify resistance-determining mutations in the voltage-gated sodium channel gene, genomic DNA from individual ticks representing both susceptible and resistant strains was used (N=10-36). Specifically, the region of domain II (S4-S5 linkers) and domain III (S6) that contain the mutations was amplified using oligonucleotide primers designed to cover all mutation regions in these domains (refer to Table S1 for details). Tick DNA barcoding was conducted on three larvae from each location using mitochondrial protein-coding gene cytochrome oxidase I (*cox*I) and mitochondrial ribosomal genes (12S and 16S rDNA). The primers and PCR conditions are indicated in Table S2.

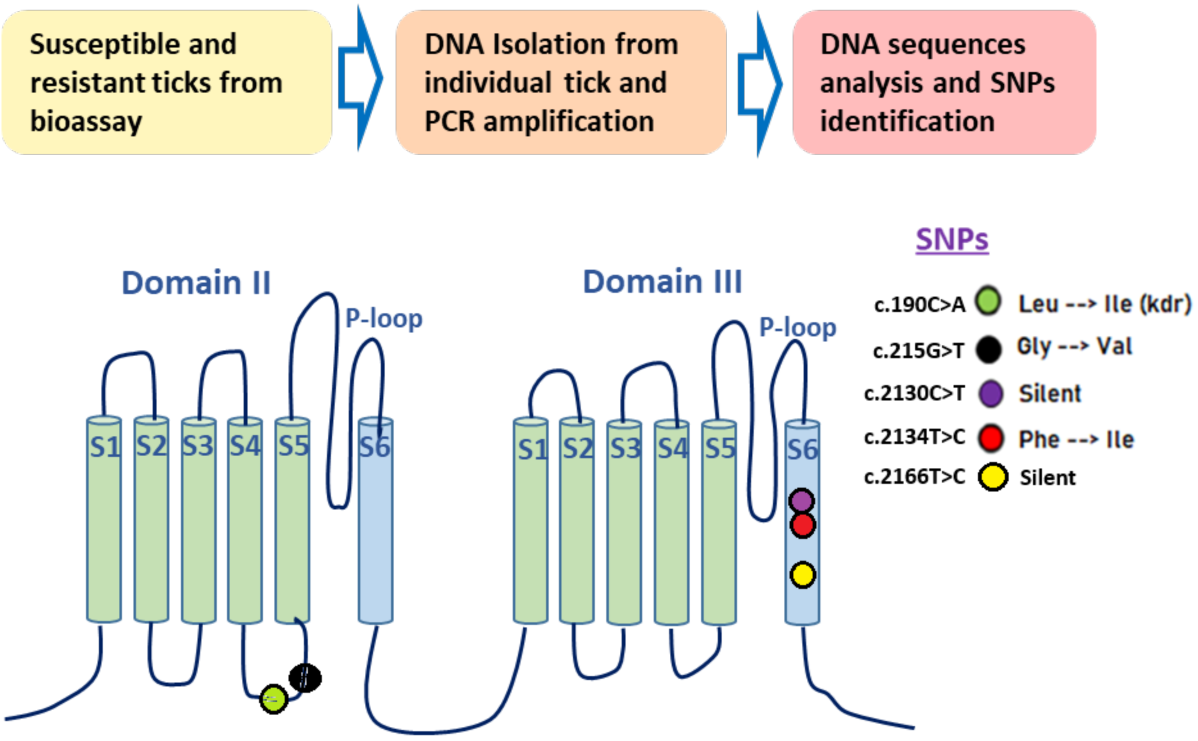

Figure S3. The location of resistant mutations (SNPs) on S4-S5 linker in Domain II (c.190C>A and c.215G>T) and S6 in Domain III (c.2134T>C and c.2166T>C).

Table S3. Gene locus, primer sequences and PCR product sizes of domain II and III of the *vgsc* gene.

Gene locus	Primer names	Sequence direction (5' -> 3')	primer length (bp)	product sizes (bp)	Primer concentration (µM)	$MgCl_2$ concentration (mM)	Annealing temperature (°C)	References
Domain II,S4-S5 linker, PCR & Sequencing	DM2F1	TACGTGTGTTCAAGCTAGC	19 bp	167 bp	0.15	2.5	56	Morgan et al. 2009
	DM2R1	ACTTTCTTCGTAGTTCTTGC	20 bp		0.15			
Domain III, First round PCR	DM3F1	AAGAGGACCAACCGGAATACG	21 bp	135 bp	0.15	2.5	56	Stone et al. 2014
	DM3R1	TCTTCTTTTGTTCATTGAAATTGT	24 bp		0.15			
Domain III, Second round PCR	DM3F2	acccaactgaatagagagcAAGAGGACCAACCGGAATACG	40 bp	173 bp	0.15	2.5	68	
	DM3R2	acgcacttgacttgtcttcTCTTCTTTTGTTCATTGAAATTGT	43 bp		0.15			
Domain III, Sequencing	DM3Fseq	acccaactgaatagagagc	19 bp	173 bp	1.0	2.0	50	BigDye® Terminator Ready Reaction Premix (ABI)
	DM3Rseq	acgcacttgacttgtcttc	19 bp		1.0			

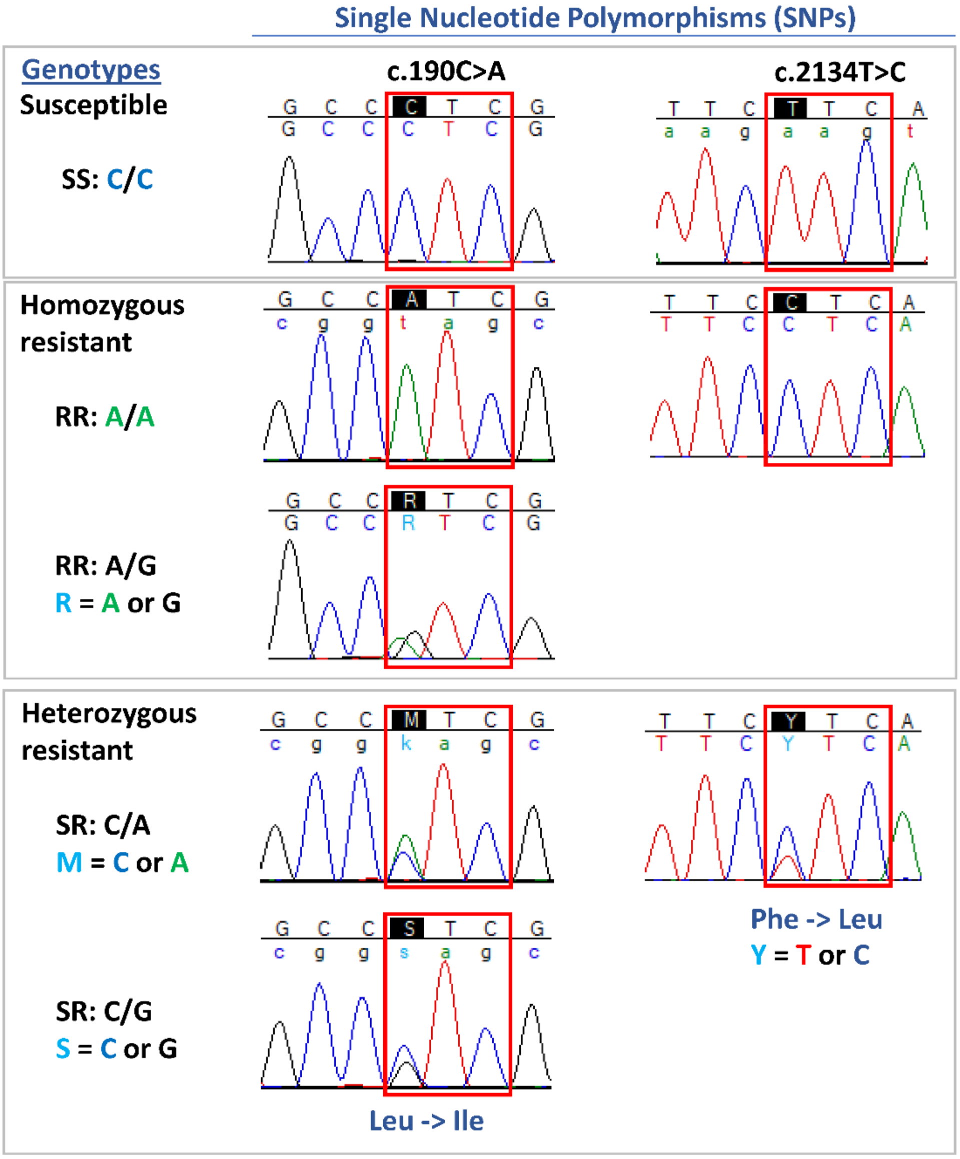

Figure S4. A representative chromatogram of DNA sequences at specific mutation sites. Red boxes are used to mark translation codons, which include SNPs that will change the amino acid composition of the protein. The characteristic double peak of a heterozygous mutation is clearly visible in the chromatogram from Sanger sequencing.

RFLP: Domain II

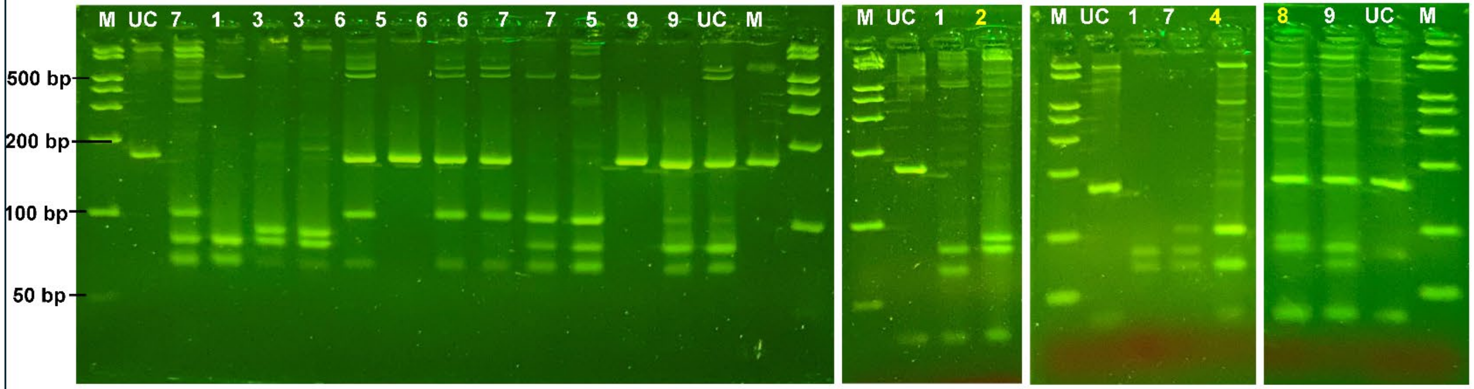

No.	Genotypes c.190C>A	Genotypes c.215G>T	RFLP Fragments (bp)
1	SS	SS	77, 69, 21
2	SS	RR	90,77
3	SS	RS	90, 77, 69, 21
4	RR	SS	98, 69
5	RR	RR	167
6	RR	RS	167, 98, 69
7	RS	SS	98, 77, 69,21
8	RS	RR	167, 90, 77
9	RS	RS	167, 98, 90 77, 69, 21
**UC**			**Uncut**

RFLP: Domain III

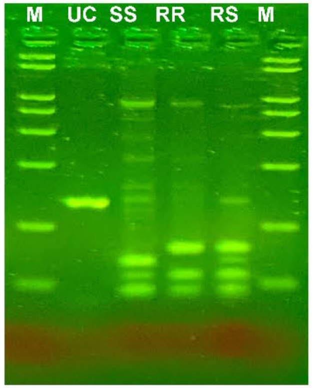

**Genotypes** c.2134T>C	**RFLP fragments (bp)**
Uncut (UC)	135
SS	67/56/47
RR	79/56/47
RS	79/67/56/47

Figure S5. RFLP patterns were observed when the PCR products of Domain II (167 bp) and Domain III (135 bp) were cut with BsaJI and MboII RE enzyme, respectively. These differentiated patterns were identified using gel electrophoresis, with an accompanying table indicating fragment sizes for each genotype. The tables demonstrate the expected fragment patterns from the PCR products cleaved by BsaJI or MboII enzymes, corresponding to each genotype of Domain II and III, respectively. M= DNA marker. The expected 21 bp product was not clearly visible on the gel, appearing very faintly below the 50 bp DNA marker.

Table S4. Resistance allele frequency of c.170T>C in domain II and c.2166T>C in domain III.

Locations	RR values	N	c.170T>C (super-kdr)				N	c.2166T>C (synonymous mutation)			
			SS	RR	RS	RAF		SS	RR	RS	RAF
OSU, susceptible	-	10	10	0	0	0	10	10	0	0	0
Lamphun	1.00 (0.62, 1.60)	6	6	0	0	0	0	-	-	-	-
Ratchaburi	1.39 (0.83, 2.31)	4	3	0	1	12.5	2	0	0	2	50.0
Chiangrai	1.60 (0.84, 3.05)	10	10	0	0	0	10	3	3	4	50.0
Ayuthaya	1.73 (0.97, 3.08)	7	7	0	0	0	7	0	0	7	50.0
Surat thani	2.50 (1.23, 5.08)	1	1	0	0	0	1	0	0	1	50.0
Lopburi	2.85 (1.07, 7.56)	6	6	0	0	0	3	1	0	2	33.3
Phayao	4.79 (0, 4.96E+31)	2	2	0	0	0	2	0	0	2	50.0
Nakhonprathom	9.15 (4.52, 18.00)	7	7	0	0	0	6	2	1	3	41.7
Petchabun	13.00 (7.83, 23.00)	13	13	0	0	0	10	3	2	5	45.0
Krabi	16.00 (11.00, 24.00)	10	10	0	0	0	10	10	0	0	0
Chachoengsao	17.00 (11.00, 27.00)	3	3	0	0	0	7	2	0	5	35.7
Chanthaburi	20.00 (12.00, 32.00)	21	21	0	0	0	21	10	2	9	31.0
Chumphon	27.00 (17.00, 43.00)	17	11	0	6	17.6	12	5	2	5	37.5
Chonburi (Bang Lamung)	NA	30	30	0	0	0	29	8	8	13	50.0
Chonburi (Ban Chang)	NA	33	33	0	0	0	33	1	21	11	80.3
Bangkok-2 (Phasichareon)	56.00 (36.00, 87.00)	30	30	0	0	0	29	0	11	18	69.0
Total		210					192				

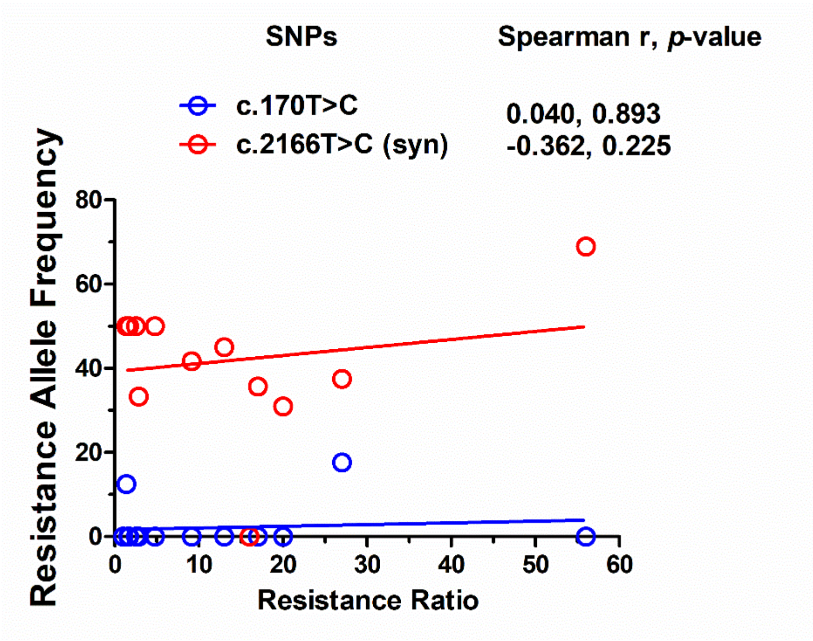

Figure S6. Correlation between Resistance allele frequency (RAF) and Resistance Ratio (RR) values of SNPs at c.170T>C and c.2166T>C. The RAF values of both mutations did not correlate well with RR values.

Table S5. The genotypes of engorged females and their offspring. New alleles (* = c.190CG, ** = c.190AG) were detected among these samples.

Provinces	Stage	N	c.190C>A (*kdr*)				c.215G>T			c.2134T>C		
			SS	RR	RS		SS	RR	RS	SS	RR	RS
Bangkok (Talingchan)	Mother	1	1	0	0		1	0	0	0	0	1
	Offspring	35	8	0	27		8	0	27	32	0	3
Krabi	Mother	1	1	0	0		1	0	0	0	0	1
	Offspring	10	10	0	0		10	0	0	4	1	5
Chumphon	Mother	1	0	0	1		1	0	0	0	0	1
	Offspring	36	8	10	18		21	3	12	22	1	13
Petchabun	Mother	1	0	0	1		0	0	1	0	0	1
	Offspring	28	9	5	14		9	5	14	8	0	20
Chiangrai	Mother	1	1	0	0		1	0	0	1	0	0
	Offspring	10	10	0	0		6	0	4	10	0	0
OSU, Rh. sanguineus (temperate lineage)	Mother	1	1	0	0		1	0	0	1	0	0
	Offspring	10	9	0	1		9	0	1	10	0	0
Nakhonprathom	Mother	1	0	1	0		0	1	0	1	0	0
	Offspring	16	1	7	8		1	7	8	6	0	10
Suratthani	Mother	1	0	0	1		1	0	0	1	0	0
	Offspring	10	3	1	6		4	0	6	5	0	5
Ayuthaya	Mother	1	0	0	1		0	0	1	1	0	0
	Offspring	16	7	1	8		6	1	9	16	0	0
Phayao	Mother	1	0	0	1		0	0	1	0	0	1
	Offspring	30	16	0	14		16	0	14	20	0	10
Chachoengsao	Mother	1	0	0	1		0	0	1	1	0	0
	Offspring	30	9	4	17		9	4	17	19	2	9
Lopburi	Mother	1	0	1	0		0	1	0	1	0	0
	Offspring	30	5	0	25		5	6	19	23	0	7
Lamphun	Mother	1	1	0	0		1	0	0	1	0	0
	Offspring	32	32	0	0		28	4	0	32	0	0
Nakhonprathom	Mother	1	0	1	0		0	1	0	1	0	0
	Offspring	16	1	7	8		1	7	8	6	0	10
Bangkok (Phasi Charoen)	Mother	1	0	1**	0		0	0	1	1	0	0
	Offspring	31	0	31**	0		0	13	18	31	0	0
Chonburi (Bang Lamung)	Mother	1	0	0	1		0	0	1	0	0	1
	Offspring	30	7	8	15*		9	8	13	8	7	15
Chonburi (Ban Chang)	Mother	1	0	1	0		0	0	1	1	0	0
	Offspring	33	0	31**	2*		1	21	11	31	0	2

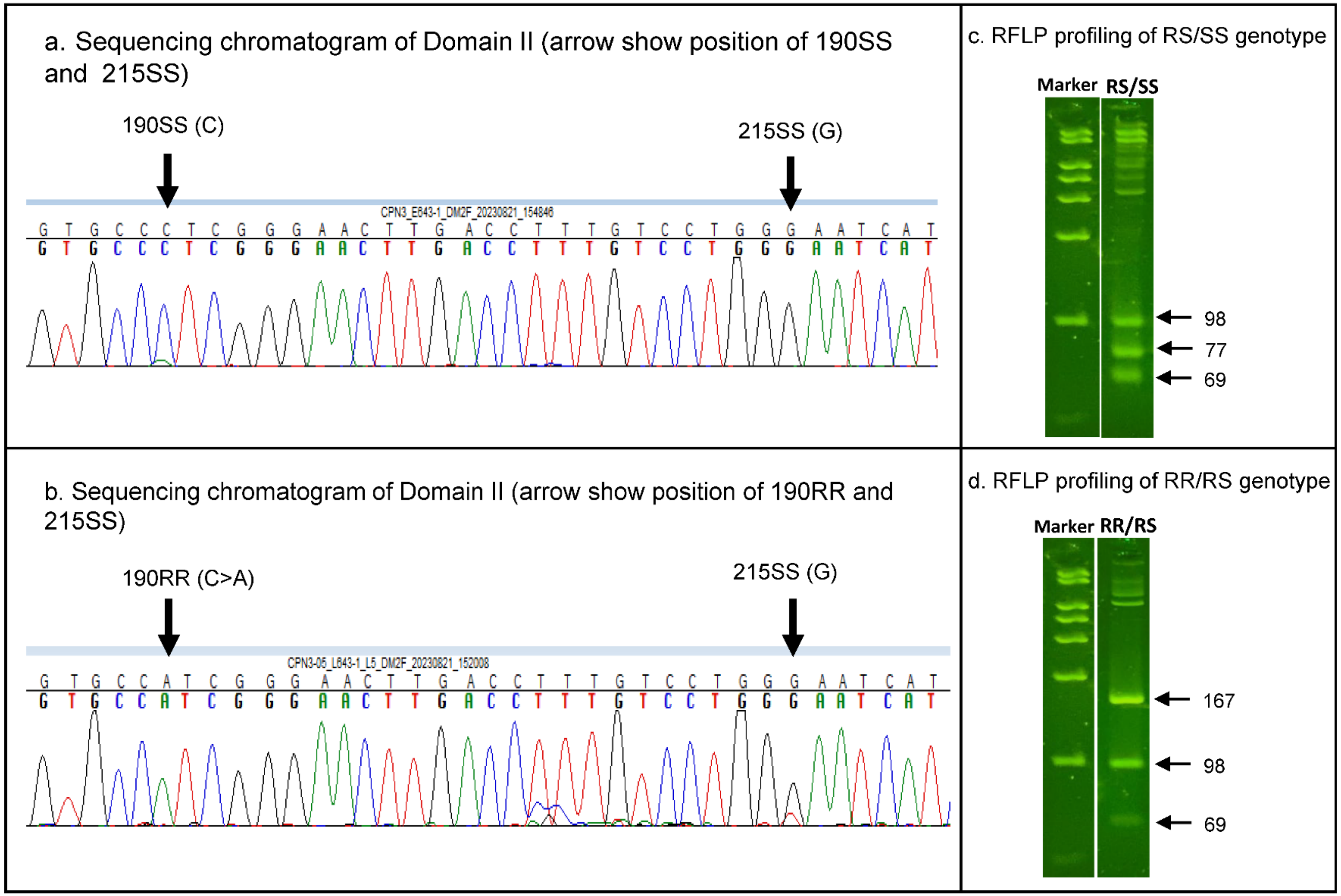

Figure S7. The PCR-RFLP assay validation results showed discrepancies between DNA sequencing and PCR-RFLP outcomes for two samples. The raw sequencing chromatograms indicated minor peaks close to background noise levels.

Four hundred seventy-three larvae from 19 locations (16 provinces in Thailand) were genotyped by DNA sequencing or Restriction fragment length polymorphism (RFLP) as detailed in the below Table S6.

Table S6. The number of larvae from each location that has undergone genotyping by DNA sequencing and PCR-RFLP assay as detailed below.

**Location**	**Genotyping by**		**Total**
	**DNA sequencing**	**PCR-RFLP**	
Ayuthaya	7	9	16
Bangkok-2 (Phasichareon)	31	0	31
Bangkok (Talingchan)	0	35	35
Chacheungsao	9	21	30
Chiangrai	10	0	10
Chonburi (Ban Chang)	33	0	33
Chonburi (Bang Lamung)	30	0	30
Chumphon	19	17	36
Lumphun	6	26	32
Lop Buri	9	21	30
Nakhon Prathom	7	9	16
Petchabun	13	15	28
Prachinburi	0	35	35
Surat Thani	1	9	10
Chanthaburi*	21	0	21
Krabi	10	0	10
Phayao	2	28	30
Ratchaburi	4	26	30
susceptible, OSU-USA	10	0	10
**Total**	**222**	**251**	**473**

Note: (*), for ticks from Chanthaburi province, 6 larvae did not have complete sequence data for both Domain II and III, therefore the total number for some results only had N=467.

Table S7. Primer sequences and PCR product sizes of 12S, 16S rRNA genes and cytochrome oxidase subunit I (*Cox*I) gene.

**Genes**	**Primer names**	**Sequence direction (5' □ 3')**	**primer length (bp)**	**product size (bp)**	**Primer concentration (µM)**	**$MgCl_2$ concentration (mM)**	**Annealing temperature (°C)**	**References**
12S rDNA	T1B	AAACTAGGATTAGATACCCT	204.	370	0.175	2.5	57	Beati L, 2001
	T2A	AATGAGAGCGACGGGCGATGT	21		0.175			
16S rDNA	mt-rrs1	CTGCTCAATGATTTTTTAAATTGCTGTGG	29	445	0.175	2.5	58	Ushijima, 2003
	mt-rrs2	CCGGTCTGAACTCAGATCAAGTA	23		0.175			
*Cox*I	Cox1F	GGAACAATATATTTAATTTTTGG	23	820	0.2	2.5	55	Chitimia, 2010
	Cox1R	ATCTATSCCWACTGTAAATATATG	24		0.2			

4. Population genetics of *Rhipicephalus linnaei* tick populations in Thailand.

Engorged female ticks were collected from domestic canines across Thailand and transported to WRAIR-AFRIMS for the insecticide resistance bioassay and genotyping. Sequences were aligned and compared with sequences retrieved from the GenBank database using MEGA 7 or 10. The distance matrix was calculated using MEGA 7. Results revealed that genetic similarities among Thai populations are 99.6-100% for COXI, 100% for 16S rDNA and 99.6-100% for 12S rDNA. Thai populations were genetically distinct from the USA laboratory population, with 83.6-84.3%, 87.9%, 96.9% genetic similarity for COXI, 12S and 16S rDNA, respectively (Fig. S6-S8).

A population genetic analysis of *Rh. linnaei* OSU-USA laboratory population (Pop 1) and Thai populations (Pop 2) was performed using the STRUCTURE software with COXI sequences (N=64). The data showed there is no population structure among Thai tick populations but there is clear genetic differentiation between USA and Thai populations (deltaK=2) with 100% probability for every tested individual grouped to the respective inferred clusters.

Abbreviations for locations: AYA= Ayutthaya, BKK=Bangkok, CBI=Chonburi CCO=Chachoengsao, CRI=Chiangrai, CPN=Chumphon, CTI=Chantaburi, KBI=Krabi, LRI=Lop Buri, LPN=Lamphun, NPT=Nakhon Prathom, PRI=Prachinburi, PNB=Petchabun, RBR=Ratchaburi, SNI=Surat Thani.

Reference:

- MEGA7: Molecular Evolutionary Genetics Analysis version 7.0 for bigger datasets Kumar S, Stecher G, and Tamura K (2016) Molecular Biology and Evolution 33:1870-1874

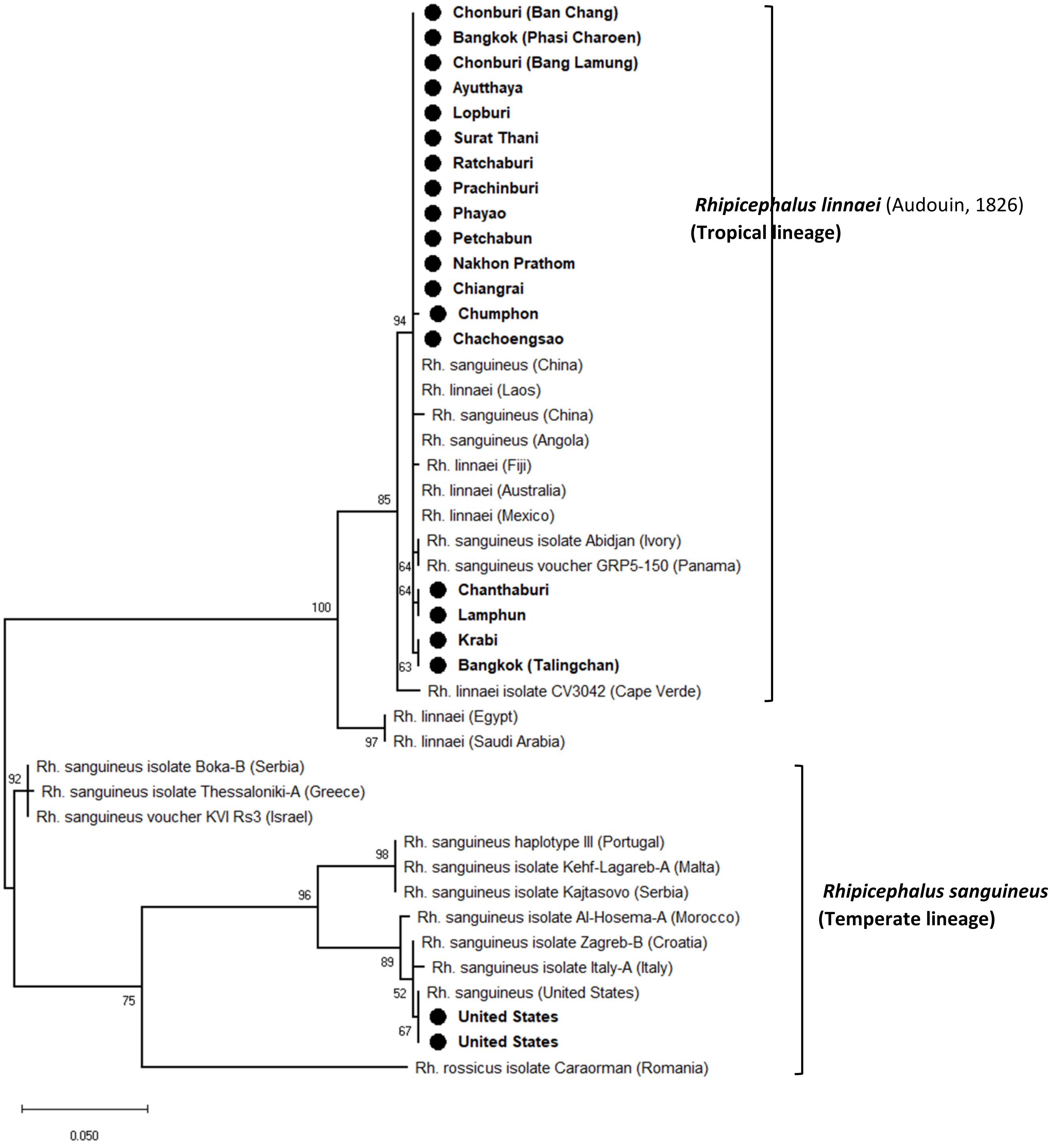

Figure S8. Phylogenetic analysis of the relationship of cytochrome oxidase subunit I (*cox*I) gene between *Rh. linnaei* of Thai strains and populations from populations from other countries using the T92+G model with 1,000 bootstraps value on each node (only >50% bootstrap is shown) in MEGA 7 software. Sequences from this study (✱) and sequences retrieved from Genbank database starting with accession numbers.

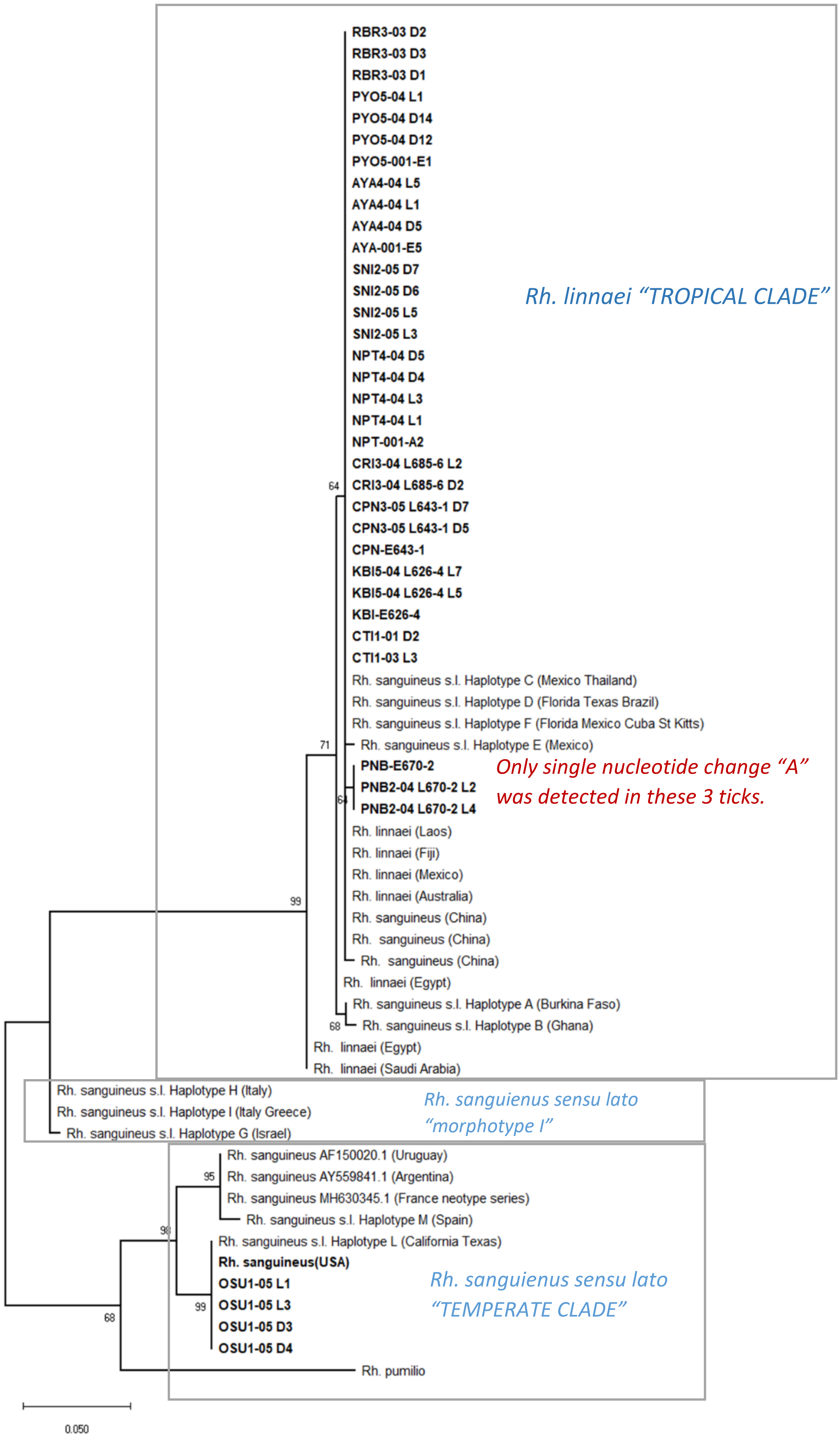

Figure S9. 12S rDNA gene. Sequence similarity shows 99.6-100% identity among Thai populations and 87.9% identity between Thai (bold letters) and USA populations (OSU-USA).

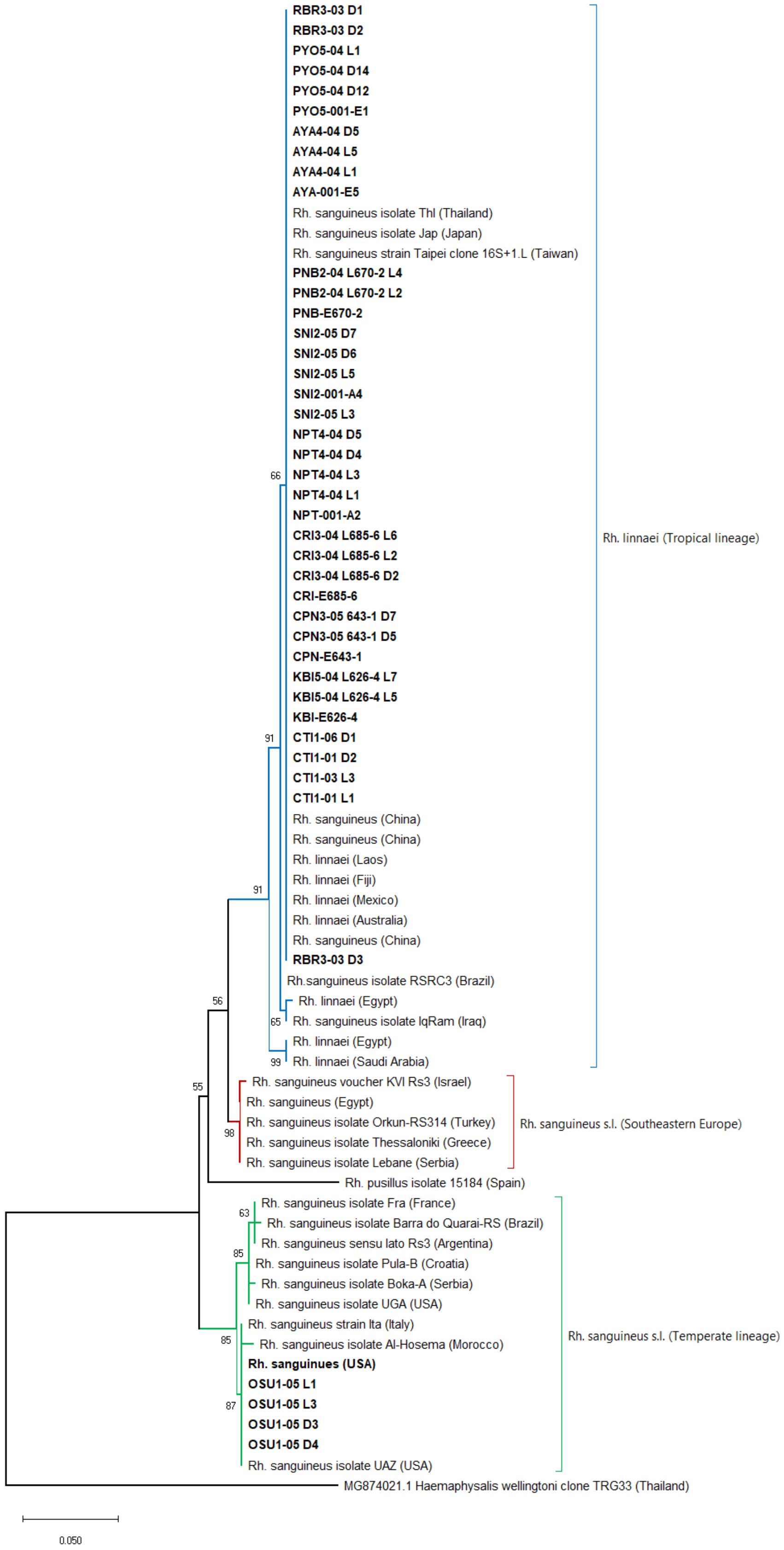

Figure S10. 16S rDNA (100% identity among Thai strains and 96.9% identity between Thai (bold letters) and USA populations (OSU).

DNA sequence: Cytochrome oxidase subunit I (*COXI*)
N=64 sequences (Thai=59, USA=5)
STRUCRURE parameters: data were run with K (1-8) with 10,000 burn-in period, 50,000 repeat MCMC, and 20 iterations. Using default parameters: Admixture,

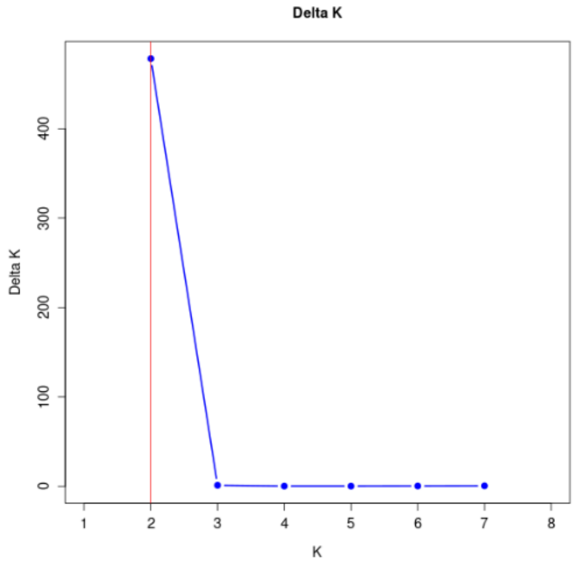

Evanno method

— Evanno G, Regnaut S, Goudet J (2005) Detecting the number of clusters of individuals using the software structure: a simulation study. *Molecular Ecology*, 14:2611–2620. [link]

DeltaK:

K	Reps	Mean LnP(K)	Stdev LnP(K)	Ln'(K)	|Ln''(K)|	Delta K
1	20	-1356.31500	0.51736	NA	NA	NA
2	20	-161.07000	2.40943	1195.24500	1152.66500	478.39674
3	20	-118.49000	28.15117	42.58000	32.42000	1.15164
4	20	-108.33000	29.70212	10.16000	6.31500	0.21261
5	20	-104.48500	35.74212	3.84500	7.17000	0.20060
6	20	-93.47000	36.20516	11.01500	12.45000	0.34387
7	20	-94.90500	31.68867	-1.43500	16.29500	0.51422
8	20	-80.04500	30.52734	14.86000	NA	NA

K selection was acquired from the website: https://lmme.ac.cn/StructureSelector/, using input files from the STRUCTURE result folder.

Given Pop	Inferred clusters		Number of Individuals
	1	2	
1:00	1.00	0	5
2:00	0	1.00	59

Frequency divergence among pop		
	1	2
1	-	0.4131
2	0.4131	-

Bar plot by Q-matrix from STRUCTURE software

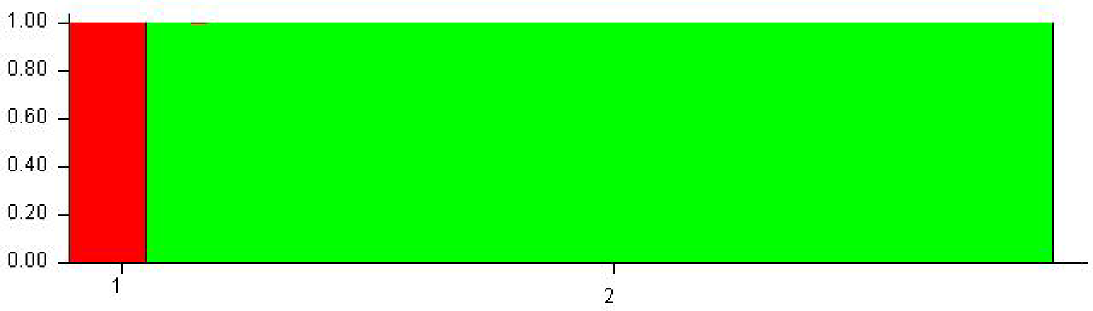

Figure S11. Population genetic analysis conducted using STRUCTURE software.
